# Multiconfigurational Calculations and Photodynamics Describe Norbornadiene Photochemistry

**DOI:** 10.1021/acs.joc.2c02758

**Published:** 2023-04-06

**Authors:** Federico J. Hernández, Jordan M. Cox, Jingbai Li, Rachel Crespo-Otero, Steven A. Lopez

**Affiliations:** † Department of Chemistry and Chemical Biology, Northeastern University, Boston, Massachusetts 02115, United States; ‡ School of Physical and Chemical Sciences, Queen Mary University of London, Mile End Road, London E1 4NS, U.K.; § Hoffmann Institute of Advanced Materials, Shenzhen Polytechnic, 7098 Liuxian Blvd, Nanshan District, Shenzhen 518055, People’s Republic of China

## Abstract

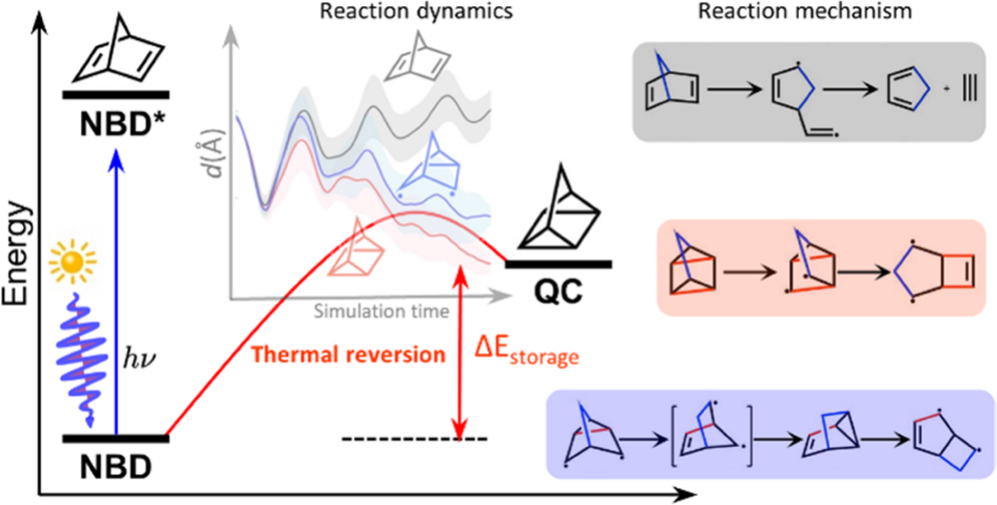

Storing solar energy is a vital component of using renewable energy sources to meet the growing demands of the global energy economy. Molecular solar thermal (MOST) energy storage is a promising means to store solar energy with on-demand energy release. The light-induced isomerization reaction of norbornadiene (**NBD**) to quadricyclane (**QC**) is of great interest because of the generally high energy storage density (0.97 MJ kg^–1^) and long thermal reversion lifetime (*t*_1/2,300K_ = 8346 years). However, the mechanistic details of the ultrafast excited-state [2 + 2]-cycloaddition are largely unknown due to the limitations of experimental techniques in resolving accurate excited-state molecular structures. We now present a full computational study on the excited-state deactivation mechanism of **NBD** and its dimethyl dicyano derivative (**DMDCNBD**) in the gas phase. Our multiconfigurational calculations and nonadiabatic molecular dynamics simulations have enumerated the possible pathways with 557 S_2_ trajectories of **NBD** for 500 fs and 492 S_1_ trajectories of **DMDCNBD** for 800 fs. The simulations predicted the S_2_ and S_1_ lifetimes of **NBD** (62 and 221 fs, respectively) and the S_1_ lifetime of **DMDCNBD** (190 fs). The predicted quantum yields of **QC** and **DCQC** are 10 and 43%, respectively. Our simulations also show the mechanisms of forming other possible reaction products and their quantum yields.

## 1 Introduction

**Figure 1:**
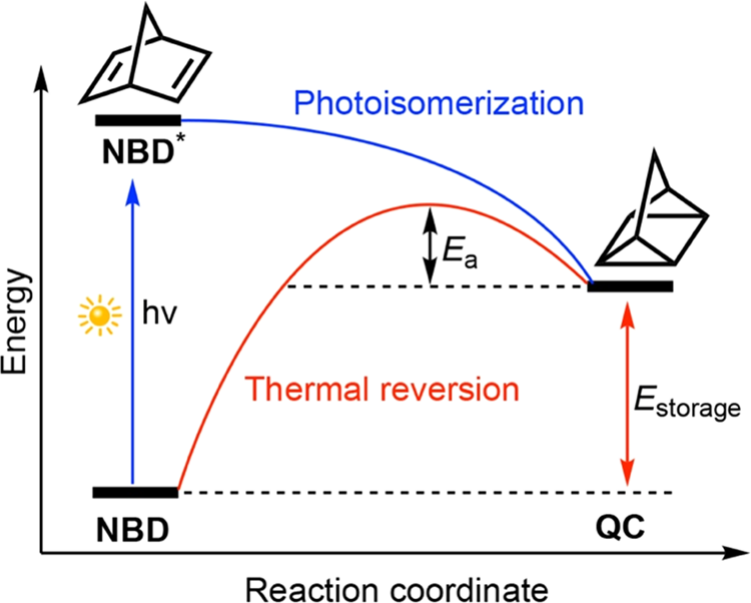
Illustration of solar thermal energy storage based on **NBD** and **QC** isomerization.

Sunlight is an essentially renewable and sustainable energy resource. The utilization of solar energy requires effective technologies to convert intermittent sunlight into steady electricity or fuel for regular usage. Efforts have been made to develop, for instance, solar cells,^1−6^ CO_2_ reductions,^7−10^ water splitting,^11,12^ energy-dense fuels,^13,14^ photoswitches,^15−17^ and solar thermal energy storage.^18^ Among various prominent technologies, molecular solar thermal (MOST) energy storage provides a promising tool to store solar energy and release it on demand.^19−21^ MOST devices are developed based on photoswitchable molecules. MOST materials isomerize from a thermodynamically stable structure to a metastable structure, storing the absorbed energy in strained chemical bonds. Many organic molecules have been investigated for MOST applications, such as azobenzene,^16,22−24^ anthracene,^25,26^ tetracarbonyl-fulvalene-diruthenium,^27−30^ dihydroazulene/vinylheptafulvene (**DHA**/**VHF**),^31−34^ bicyclooctadiene/tetracyclooctane (**BOD**/**TCO**),^35,36^ and norbornadiene/quadricyclane (**NBD**/**QC**).^37−43^ The **NBD**/**QC** system is one of the top candidates, where the **NBD** undergoes a [2 + 2]-photocycloaddition to the photoisomer **QC** (Figure 1).^44,45^

The thermal reversion of **QC** to **NBD** releases a storage energy of 88 kJ mol^–1^, corresponding to a storage density of 0.97 MJ kg^–1^.^35^ The back reaction barrier of **QC** is about 138 kJ mol^–1^,^46^ which leads to a half-life of 14 h at 140 °C (i.e., *t*_1/2,300K_ = 8346 years).^47^ Such a lifetime allows the **NBD**/**QC** system to store the accumulated solar energy effectively. However, the high energy of absorption maximum (200–230 nm^48,49^) and the modest quantum yield (QY) of **QC** (5%) could limit its efficiency for solar energy conversion.^20,41,42,50^ Many studies have shown that the functionalization of the π_CC_ bonds of NBD can red-shift the absorption and increase the QY.^37,39,40,51−54^ But the increased molecular weight of substituents reduces the energy storage density to 0.10–0.56 MJ kg^–1^.^41^ A high-throughput virtual screening studies on 3239 **NBDs**(55) and machine-learning-assisted screening of over 10^25^ substituted **NBDs** discovered only 15 candidates with absorption longer than 350 nm, storage density larger than 0.4 MJ kg^–1^, and back reaction barriers exceeding 150 kJ mol^–1^ (*t*_1/2,300K_ = 459 824 years).^56^

**Scheme 1:**
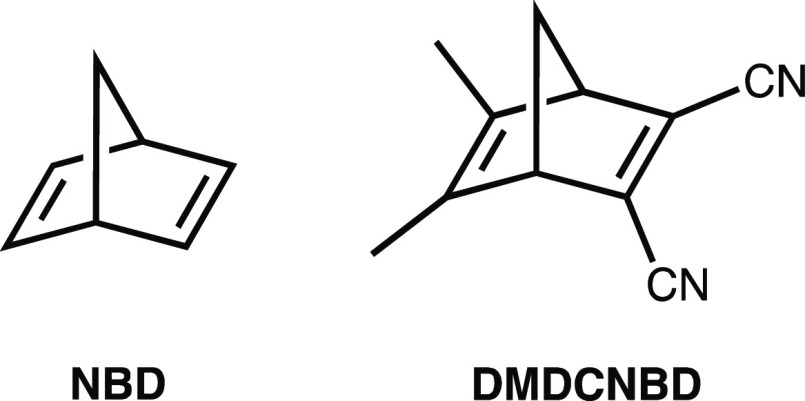
Chemical Structures of **NBD** and **DMDCNBD**

Literature reports on the photophysical properties and reaction energies provide important design features, but considerably less is known about the excited-state reaction mechanisms of **NBD** and its derivatives. The ultrafast photochemical **NBD** → **QC** interconversion has been the primary focus of computational and experimental elucidation of the low-lying absorption at 212 nm (5.85 eV) corresponding to an excitation to the 3s-Rydberg state.^49,57,58^ The mechanistic studies are limited to the exploration of the excited-state potential energy surface (PES) and characterization of the S_1_/S_0_ minimum energy conical intersection (MECI).^59^ The S_1_/S_0_ MECI shows a bond-forming geometry indicating an important role in the [2 + 2]-cycloaddition reactions. A grid-based quantum dynamics study of **NBD** observed S_1_ → S_0_ transition through the S_1_/S_0_ MECI, while the simulations were carried out considering 3 degrees of freedom.^60^ Other reports described the substituent effects on the excitation energy, excited-state minimum energy path (MEP), and the energy storage of the functionalized **NBD**.^61,62^ However, the excited-state dynamics of **NBD** that fully traverse the reaction coordinate from the initially excited **NBD** (Franck–Condon region) to product(s) have not been explored yet. In this work, we use nonadiabatic dynamics coupled with CASSCF calculations to study the deactivation pathways of photoexcited **NBD** and its dimethyl dicyano derivative **DMDCNBD** (Scheme 1). **DMDCNBD** was selected as a model system of the related compound **TMDCNBD** (Figure 9) to learn how substituents affects the photodynamics of **NBD**. **TMDCNBD** has a red-shifted absorption spectrum due to the presence of the cyano groups, and it shows higher photoisomerization QYs to **QC** (57–96%),^63,64^ suggesting that substituents might block cycloreversions and side reactions. We analyze the deactivation mechanism after photoexcitation. We also offer a mechanistic map showing other possible reaction products and their predicted quantum yields.

## 2 Computational Methods

**Figure 2:**
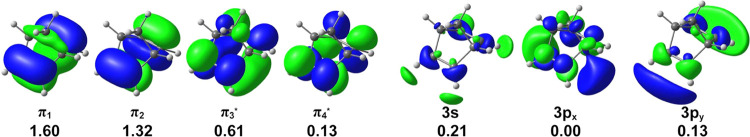
Illustration of the (4,7) space of **NBD** with the occupations averaged over six electronic states, computed with SA6-CASSCF(4,7)/ANO-S-VDZP. The isovalue is 0.03.

We selected the CASSCF method for the gas-phase excitations and prepared a suitable active space. Previous experimental and theoretical studies on **NBD** found a low-lying absorption at 212 nm (5.85 eV) corresponding to an excitation to the 3s-Rydberg state.^49,57,58^ Antol proposed a (4,5) active space of four π-electrons and four π-orbitals with restricted single excitations to an auxiliary 3s-orbital to compute the excited-state potential energy surface (PES) of **NBD**.^59^ Recently, Remacle and co-workers included another 3p-orbital forming an (8,8) space to model the quantum dynamics of **NBD** over the eight electronic states.^60^ Since we are interested in the photochemical reactions of **NBD** occurring in the low-lying states, we considered a (4,7) active space with 3s-, 3p*_x_*-, and 3p*_y_*-orbitals. The selection of these Rydberg orbitals was based on the lowest four excitations. We note a recent work by Marazzi and Pastore that used the same active space studying the excited-state PES of **NBD**.^65^ We further benchmarked the excitations of **NBD** with time-dependent density functional theory (TDDFT) using the range-separated functional, ωB97XD^66^ and equation of motion coupled cluster with singles and doubles (EOM-CCSD)^67^ and the aug-cc-pVDZ basis sets.^68^Figure 2 illustrates the (4,7) active space computed with the six-state-averaged (SA6) CASSCF(4,7)/ANO-S-VDZP method. The ANO-S-VDZP basis set^69−72^ has a quality generally comparable to aug-cc-pVDZ.^73^

**Figure 3:**
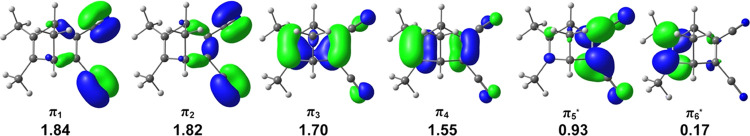
Illustration of the (8,6) space of **DMDCNBD** with the occupations averaged over six electronic states, computed with SA6-CASSCF(8,6)/ANO-S-VDZP. The isovalue is 0.03.

The active space for **DMDCNBD** contains the four π-electrons and four π-orbitals involved in bond formations and additional four π-electrons and two π-orbitals of the cyano groups, forming an (8,6) active space. The selection of π-orbitals (Figure 3) was based on the TDDFT results at the ωB97XD/aug-cc-pVDZ level of theory.

The geometries of **NBD** and **DMDCNBD** were optimized with SA6-CASSCF(4,7)/ANO-S-VDZP and SA6-CASSCF(8,6)/ANO-S-VDZP, respectively. Hessian calculations confirmed the local minima with no imaginary frequencies. We assessed the quality of CASSCF excitation energies with the extended multistate complete active space second-order perturbation (XMS-CASPT2) method. We used OpenMolcas 19.11 in the CASSCF and XMS-CASPT2 calculations.^74^ The TDDFT and EOM-CCSD calculations were performed with ORCA 4.2.0.^75^

The gas-phase photodynamics simulations used Tully’s surface hopping method based on the product of the time-independent nonadiabatic couplings (NACs) and velocities.^76,77^ We applied phase corrections at every timestep based on the NAC overlaps between two consecutive time steps.^78^ To accelerate the simulations, we only considered the NACs between adjacent states and assumed zero coupling between nonadjacent states (e.g., states 1 and 3). The simulations ran in the microcanonical ensemble (NVE) and the simulation times are 500 and 800 fs for **NBD** and **DMDCNBD** with a timestep of 0.5 fs, respectively. The surface hopping calculations integrated the nuclear amplitude with a step size of 0.02 fs (i.e., 25 substeps). We applied an energy-based decoherence correction of 0.1 hartree to the nuclear amplitude.^79^ At surface hopping event, the momenta were rescaled isotropically to ensure energy conservation. The **NBD** trajectories started from the S_2_-state, whereas the **DMDCNBD** trajectories started from the S_1_-state.

800 initial conditions of **NBD** and **DMDCNBD** were sampled for gas-phase photodynamics simulations using the Wigner sampling at the zero-point energy level.^80 ,81^ The trajectories that failed to converge the CASSCF calculations were removed from the analysis. The final number of trajectories was determined by ensuring (1) a converged QY prediction, and (2) the QY values were higher than the margin of error at the 95% confidence level (Figure S4). We also computed the vertical excitation energies of the Wigner-sampled initial conditions of **NBD** and **DMDCNBD** to simulate the gas-phase absorption spectra. The computed wavelengths were expanded with narrow Gaussian functions (i.e., full width at half-maximum of 8 nm) and scaled with the oscillator strengths.

## 3 Results and Discussion

Table 1 summarizes the computed vertical excitations of **NBD** and the reported experimental results.

**Table 1:** Vertical Excitation Energies with the Principal Electronic Configurations of **NBD** Computed with the Relaxed Geometry in the Ground State

	S_1_		S_2_		S_3_	
TD-ωB97XD/aug-cc-pVDZ	5.45	π_2_ → π_3_*	6.20	π_2_ → 3s*	6.22	π_1_ → π_3_*
						π_2_ → π_4_*
EOM-CCSD/aug-cc-pVDZ	5.62	π_2_ → π_3_*	5.99	π_2_ → 3s	6.44	π_2_ → 3p_y_*
SA6-CASSCF(4,7)/ANO-S-VDZP	6.99	π_2_ → π_3_*	7.58	(π_2_ → π_3_*)^2^	7.61	π_2_ → 3s
XMS(6)-CASPT2(4,7)/ANO-S-VDZP	5.50	π_2_ → π_3_*	6.68	π_1_ → π_3_*	7.39	π_2_ → 3s
XMS(10)-CASPT2(4,7)/aug-cc-pVDZ	4.70	π_2_ → π_3_*	5.93	π_2_ → 3s	6.40	π_2_ → 3p_y_*
exp^a^	5.25	π_2_ → π_3_*	5.85	π_2_ → 3s	6.00	π_1_ → π_3_*

a Experimental data and configurations are obtained from ref (57). Energies are in eV. For convenience, the π_2_ → π_3_*, π_1_ → π_3_*, and (π_2_ → π_3_*)^2^ configurations are termed ππ*, ππ_2_*, and doubly excited (DE), respectively.

The TD-ωB97XD/aug-cc-pVDZ and EOM-CCSD/aug-cc-pVDZ calculations agree that the S_1_ and S_2_ are ππ* and 3s-Rydberg states, respectively. The TD-ωB97XD/aug-cc-pVDZ calculation predicts a mixture of ππ* and ππ_2_* states in S_3_, whereas the EOM-CCSD/aug-cc-pVDZ calculation indicates a 3p*_y_*-Rydberg state. This difference suggests a competing contribution of the valence and Rydberg excitations above 6 eV. The SA6-CASSCF(4,7)/ANO-S-VDZP calculations overestimate the excitation energies, as expected. They agree with the XMS(6)-CASPT2(4,7)/ANO-S-VDZP calculation and experimental results that S_1_ is a ππ* state and also suggest that the S_2_ is a doubly excited (DE) ππ* state, 0.03 eV lower than the S_3_, the 3s-Rydberg state. The XMS(6)-CASPT2(4,7)/ANO-S-VDZP results confirm that the doubly excited (DE) state is a higher state (S_4_). However, XMS(6)-CASPT2(4,7) predicts the S_2_ to be a second ππ* state (ππ_2_*), which overestimates the energy of the 3s-Rydberg state (7.39 eV) compared to the experimental results (5.85 eV). The overestimation is caused by an insufficient diffuseness of the ANO-S-VDZP basis set. For instance, the XMS(10)-CASPT2(4,7) calculations with aug-cc-pVDZ predict that the S_1_ and S_2_ are ππ* (4.70 eV) and 3s-Ryberg states (5.93 eV), respectively, in line with the experiments. Similar results are reported by Marazzi and Pastore using a large ANO-L basis set.^65^ However, these methods lead to a considerably higher computational cost for the dynamics simulations. Therefore, we used SA6-CASSCF(4,7)/ANO-S-VDZP for the spectrum and dynamics simulations as a compromise between computational cost and quality.

**Figure 4:**
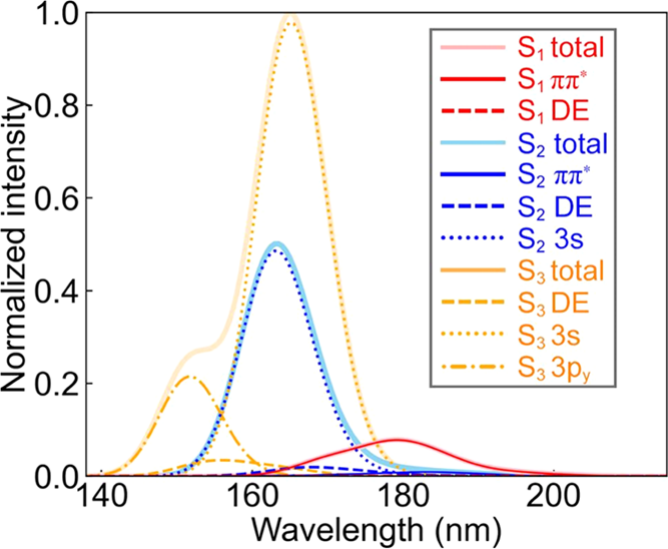
Simulated absorption spectrum for the first three bands of **NBD**. 800 structures were used to compute vertical excitation energies with SA6-CASSCF(4,7)/ANO-S-VDZP. The absorption bands are decomposed according to their excited-state electronic configurations (DE indicates a doubly excited ππ* configuration).

We computed the gas-phase vertical excitation energies of 800 Wigner-sampled **NBD** structures to explore the nature of electronic excitations to the Franck–Condon (FC) regions. Figure 4 illustrates a convoluted spectrum with contributions from various excited-state electronic configurations.

The SA6-CASSCF(4,7)/ANO-S-VDZP calculations predict a broad S_1_ absorption peak in 160–200 nm, centered at 180 nm, in line with the experimental report by Fuß et al.^49^ S_1_ is dominated by a ππ* state (94%) mixed with a minor doubly excited (DE) state (6%). The computed intensity of absorption to S_1_ is nonzero, suggesting that the ππ* state is accessible from the nonequilibrium **NBD** geometries. The S_2_ and S_3_ peaks overlap from 150–180 nm. They show strong absorption intensities corresponding to the 3s-Rydberg state. The 3s-Rydberg state in S_2_ is 40%, and the competing DE and ππ* states are 46 and 14%, respectively. However, the DE and ππ* intensities are considerably lower than the 3s-Rydberg state (Figure 4). S_3_ is composed of the 3s-Rydberg state (60%), DE state (27%), and 3p*_y_*-Rydberg state (12%). The 3p*_y_*-Rydberg state leads to a shoulder at 150 nm. The simulated spectrum for S_2_ qualitatively agrees with the experiments by Fuß and co-workers, where the irradiation populates both the 3s-Rydberg and the ππ* states.^49^

**Figure 5:**
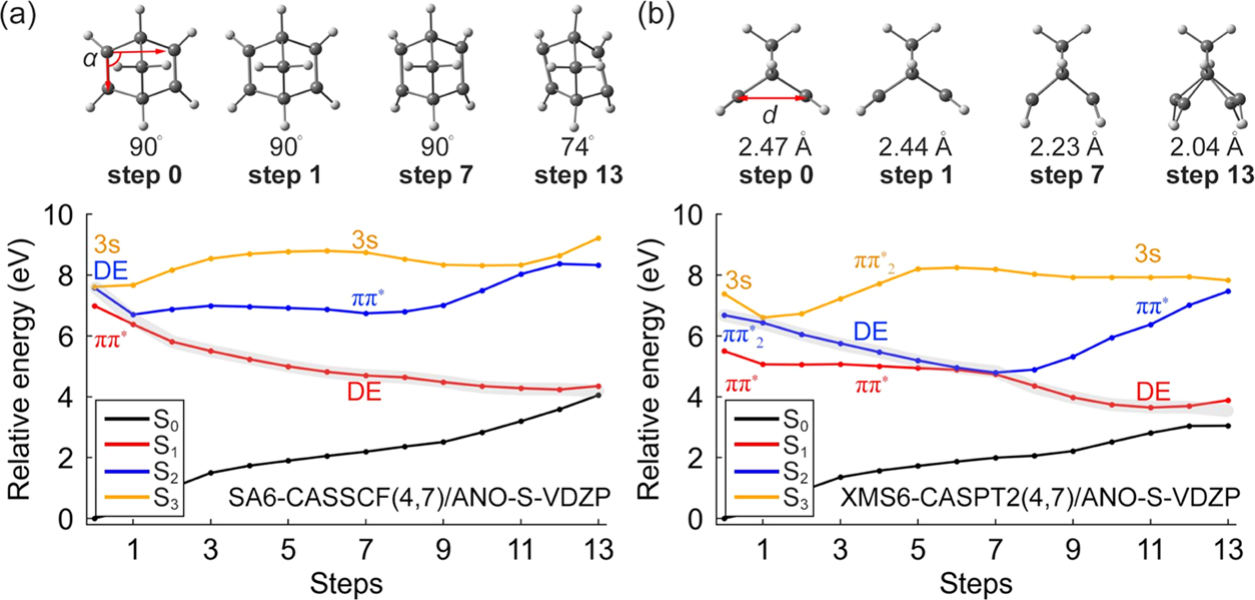
Minimum energy paths of **NBD** optimized with the SA6-CASSCF(4,7)/ANO-S-VDZP (a) and corrected with the XMS(6)-CASPT2(4,7)/ANO-S-VDZP (b). The minimization path follows the S_1_ toward the S_1_/S_0_ MECI. The gray curves highlight possible S_2_ relaxation pathways.

The minimum energy path (MEP) in Figure 5 displays the steepest descent pathways from the FC point of **NBD**, indicating a pseudo-dominant path toward mechanistic critical points (e.g., minimum energy conical intersection). Following the S_3_ and S_2_ states, the MEPs quickly converged on an excited-state local minimum (Figures S1 and S2). In contrast, the MEP from the S_1_-FC point provides an informative description of the nonradiative [2 + 2]-cycloaddition pathway from **NBD** to **QC**. Figure 5a,b compares the S_1_ MEP optimized with the SA6-CASSCF(4,7)/ANO-S-VDZP method and those corrected with XMS(6)-CASPT2(4,7)/ANO-S-VDZP.

We identified two parameters to quantify the structural changes along the reaction coordinate: a rhomboidal angle among the bond-forming carbon, *α*, and a distance between the π_CC_ bonds, *d* (Figure S3). In the S_1_-FC region, *d* and *α* are 2.47 Å and 90°, respectively. Figure 5a shows that the MEP along the S_1_ slightly decreases the π_CC_-distance (Δ*d* = 0.03 Å) at step 1, which switches the state order between the ππ* and the DE states. In the following steps, the DE state dominates the S_1_ state. The geometrical parameters resemble a rhomboidal shape (*α* = 74° and *d* = 2.04 Å), consistent with the previously reported S_1_/S_0_-MECI structures by Antol.^59^ The XMS(6)-CASPT2(4,7)/ANO-S-VDZP MEP suggests the ππ* and DE states are separated by the 3s-Rydberg and ππ_2_* states at the S_1_-FC geometry (Figure 5b). The DE state appears in S_2_ at the first step as the energy decreases with the π_CC_-distance. The DE state then leads to an S_2_/S_1_ crossing point at step 7 with *d* = 2.23 Å (Figure 5b). This indicates that the SA6-CASSCF(4,7)/ANO-S-VDZP overestimates the π_CC_-distance at the S_2_/S_1_ crossing point. Nevertheless, the SA6-CASSCF(4,7)/ANO-S-VDZP and XMS(6)-CASPT2(4,7)/ANO-S-VDZP results agree that the DE state in S_2_ drives the excited-state PES to the S_1_/S_0_ crossing point for the [2 + 2]-cycloaddition of **NBD**.

**Figure 6:**
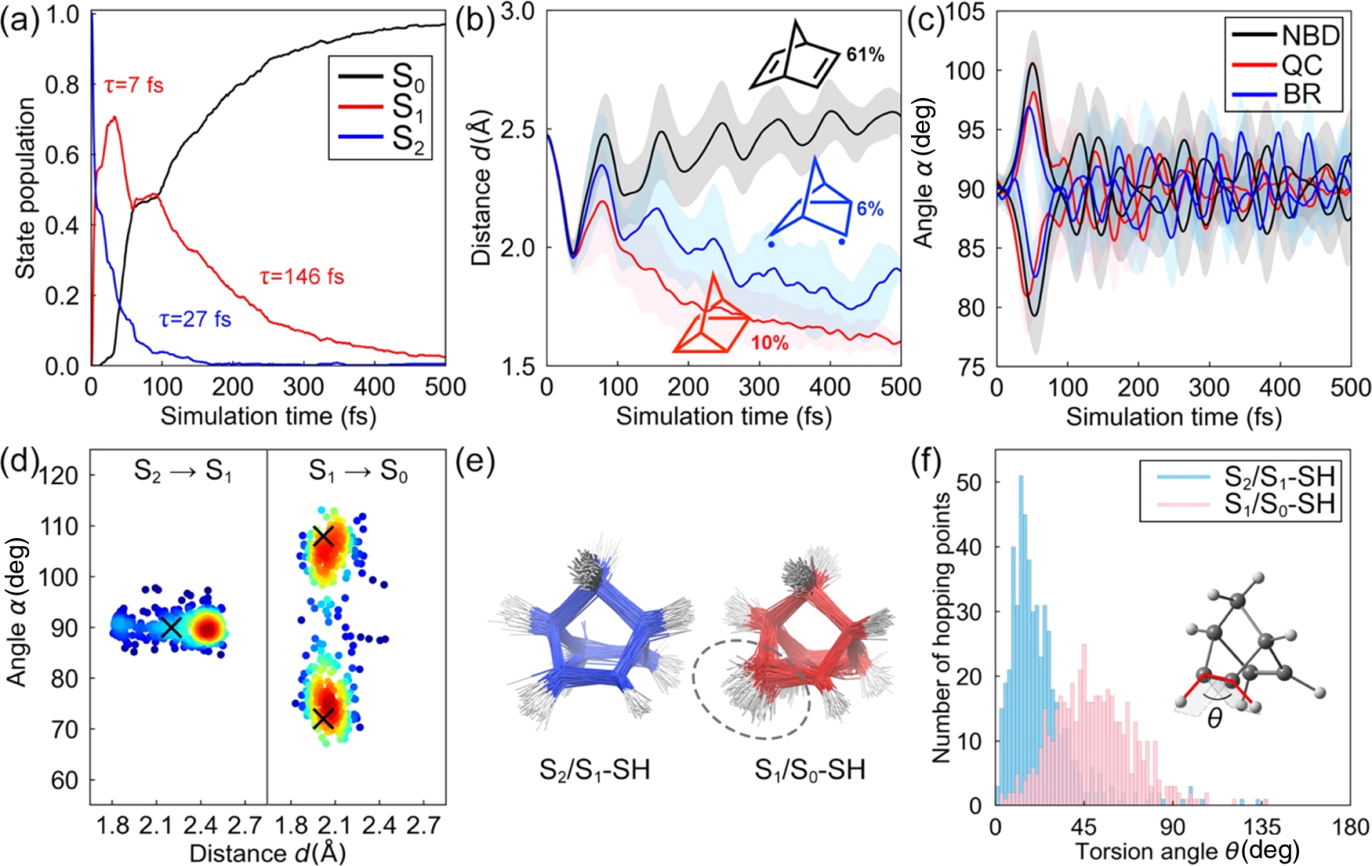
(a) State population, (b) average π_CC_-distance, (c) rhomboidal angle, and (d) surface hopping distributions of 557 **NBD** trajectories in the gas phase. In panels (b) and (c), the shaded regions render a half standard deviation to the average values to show the diversity of the trajectories. In panel (d), the “X” denotes the positions of the S_2_/S_1_ and S_1_/S_0_ MECIs. The colors from blue to red represent the accumulation of the surface hopping points from low to high, evaluated by Gaussian kernel density estimation. (e) Overlays of the 200 randomly selected S_2_/S_1_ and S_1_/S_0_ surface hopping structures. The gray circle highlights the pyramidalization of the π_CC_ bond in the S_1_/S_0_ surface hopping structures. (f) Distributions of the torsion angle θ among the S_2_/S_1_ and S_1_/S_0_ surface hopping structures. The θ values in the S_2_/S_1_ and S_1_/S_0_-MECI are 0 and 32°, respectively.

The excitation energies to the S_2_ and S_3_ states are nearly degenerate (Table 1), and their electronic excitations are mixtures of 3s-Rydberg and DE states (Figure 4). We, therefore, expect that the **NBD** trajectories starting from S_3_ would undergo rapid internal conversion to S_2_ with negligible structural changes. Hence, we started 500 fs gas-phase photodynamics simulations from S_2_. We collected 557 trajectories to obtain statistically converged results (Figures 6 and S4).

After 500 fs, 97% of the trajectories were in the S_0_ state and 2 and 1% remained in S_1_ and S_2_, respectively. Figure 6a illustrates an exponential decay of the S_2_ population with a time constant τ = 27 fs. The trajectories excited to the 3s-Rydberg state show τ = 28 fs (Figure S4a). The S_1_ (ππ* state) population increases to a local maximum of 0.71 at 32 fs (τ = 7 fs). It rises to the second maximum of 0.49 at 88 fs due to the time-discontinuous S_1_ → S_0_ decay as explained below. The overall S_1_ → S_0_ decay leads to τ = 146 fs. The previous experiment by Fuß and co-workers measured a longer time constant of the 3s-Rydberg state (τ = 420 fs) than the ππ* state (τ = 55 fs).^49^ However, our results suggest that the lifetime of the 3s-Rydberg state (τ = 27 fs) is shorter than that of the ππ* state (τ = 146 fs). The numerical difference in the lifetime may result from a local overestimation of the S_1_-PES energy near the FC region with SA6-CASSCF(4,7)/ANO-S-VDZP (compare Figure 5a,b). Such energy overestimation decreases the S_2_–S_1_ energy gap, thus accelerating the S_2_ → S_1_ decay and causing a slower S_1_ → S_0_ decay.

Figure 6b,c illustrates substantial structural changes of **NBD** during the 500 fs simulations. Structural parameters *d* and *α* have average values of 2.47 Å and 90° at the start of the trajectories, respectively. In all trajectories, the π_CC_-distance is instantaneously reduced below 2 Å within the first 50 fs (Figure 6b). At the same time, the rhomboidal angles undergo notable oscillations between 80 and 100° (Figure 6c). The trajectories then split into three pathways: (1) reversion to **NBD** (61%), (2) formation of the **QC** product (10%), and (3) formation of a singlet biradical (**BR**) intermediate (6%). The predicted QY of **QC** (10%) is in reasonable agreement with the experimental QY (5%).^20,41,42,50^ A **C**/**NBD** formation ratio of 1:6 is also predicted.

The structural changes of **NBD** originated from the nonradiative decay through the S_2_/S_1_, and subsequent S_1_/S_0_ crossing seams are depicted in Figure 6d where a scatter plot of the three-dimensional (3D) geometries of the hopping points projected onto two reaction coordinates (*d* and *α*) is shown. The majority of the S_2_/S_1_ hopping points (centered at *d* = 2.45 Å) are structurally related to those in the S_2_-FC region (centered at *d* = 2.47 Å). In contrast, a minority of those trajectories passing through the S_2_/S_1_ surface hopping points access the S_2_/S_1_-MECI (*d* = 2.20 Å and *α* = 90°) region. We interpret that the rapid S_2_ → S_1_ decay (Figure 6a) arises from the geometric proximity of the structures in the S_2_-FC region and S_2_/S_1_ crossing seam. The S_1_/S_0_ surface hopping is concentrated near *d* = 2.06 Å with a symmetric angular distribution at *α* = 74 and 105° (Figure 6d). The symmetric angular distribution indicates two equivalent S_1_/S_0_ crossing seams around the S_1_/S_0_-MECI at *d* = 2.02 Å and *α* = 72° (108°). The distinct structural differences between the S_1_/S_0_ surface hopping points and the S_2_-FC points explain the time-discontinuous S_1_ → S_0_ decay. The S_1_ → S_0_ decay depends on the π_CC_ distances and the rhomboidal angle. During the dynamics, the S_1_ → S_0_ decay stops when the S_1_ trajectories return to the FC regions with long π_CC_ distances and 90° of rhomboidal angle, e.g., at 90 fs. However, the S_2_ → S_1_ decay requires almost no geometrical changes. It constantly populates the S_1_ state, which leads to the second maximum of the S_1_ population. Figure 6e shows an overlay of the S_2_/S_1_ and S_1_/S_0_ hopping points. The S_2_/S_1_-SH structures resemble a midpoint of the linear interpolated structures between **NBD** and **QC**. However, most S_1_/S_0_-SH structures feature a pyramidalized π_CC_ bond. We quantify the extent of the pyramidalization with an HCCH torsion angle, θ. Figure 6f illustrates a histogram of θ in the trajectories to describe the π_CC_-pyramidalization distribution at the S_1_/S_0_ crossing seam. The θ ranges from 3 to 137° at the S_2_/S_1_ hopping points, but the average value is 23°. In the S_1_/S_0_-SH structures, θ ranges from 1 to 137°, and the average value increases to 49°. These results agree with prior assertions that π_CC_ pyramidalization affords easier access to S_1_/S_0_ conical intersections. Similar phenomena have been reported in the conical intersection and surface hopping structures of photoexcited conjugated molecules.^82−84^

**Figure 7:**
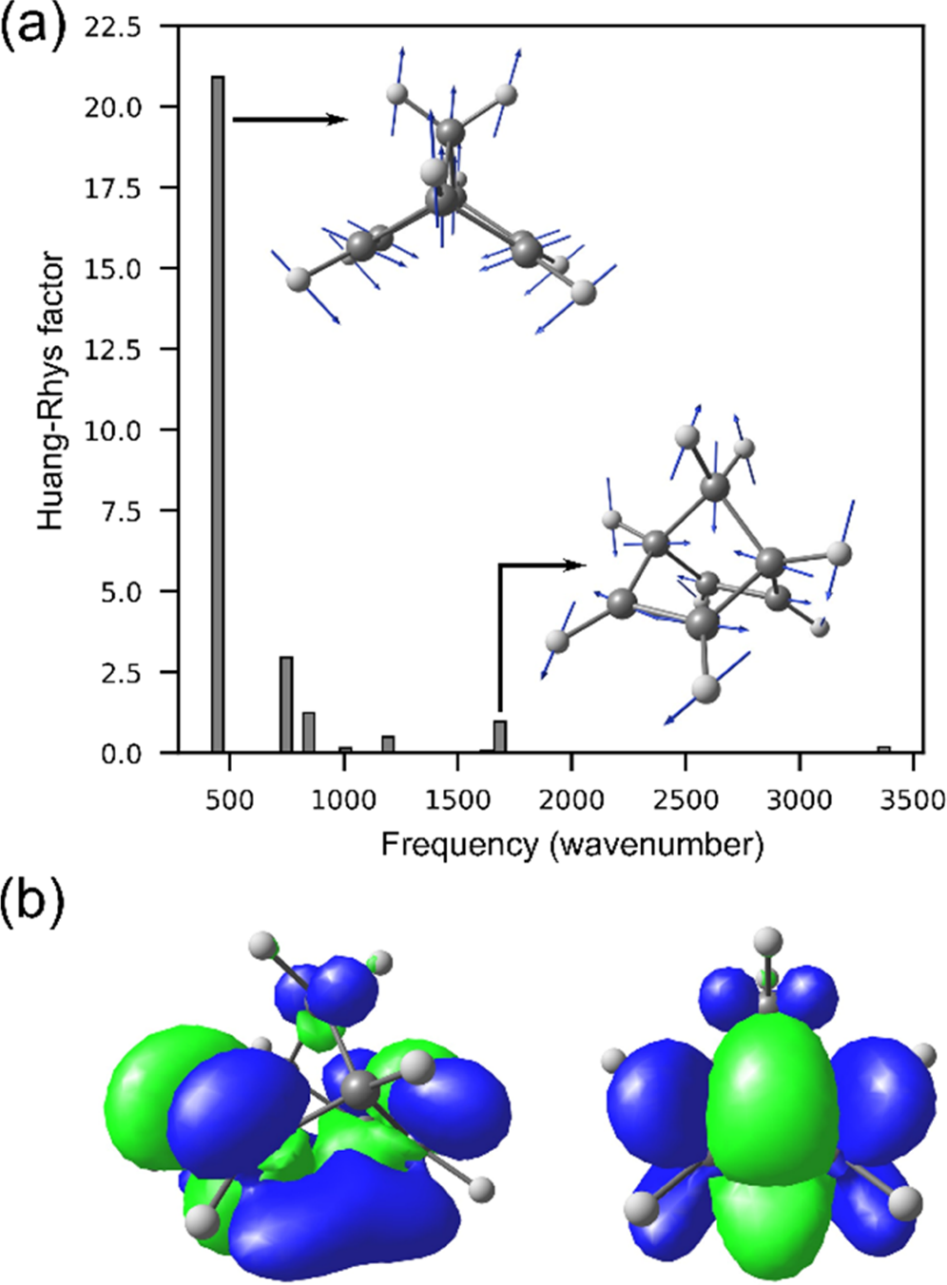
(a) Huang–Rhys factors and (b) electronic density difference map computed for the S_0_ → S_2_ transition of **NBD** at the SA6-CASSCF(4,7)/ANO-S-VDZP level of theory. In panel (b), two views of the electronic density difference map are shown. The green and blue colors represent electronic density depletion and accumulation, respectively.

The ultrafast dynamics and substantial structural changes predicted in our NAMD trajectories are in very good agreement with the experimental observations^49^ and can be interpreted in terms of an impulsive photoactivated mechanism.^85,86^ Upon photon excitation, those vibrational modes that are most coupled to the electronic excitation, i.e., showing high vibronic couplings, will take a bigger fraction of the electronic energy and therefore be activated. A Huang–Rhys factor analysis (Figure 7a) shows that, for the S_0_ → S_2_ transition, the highest vibronic coupling is predicted for the wing-flapping vibration (the two ethylenic halves moving apart). The Huang–Rhys factors were computed at the SA6-CASSCF(4,7)/ANO-S-VDZP level and using FCclasses3 software.^87^ The photoactivation of the wing-flapping vibration suggests a substantial change in *d* and *α* coordinates as observed during the first 50 fs of our NAMD trajectories (Figure 6b,c) and also in experiments.^49^ An electron density difference map, computed at the same level of theory for the S_0_ → S_2_ transition, shows that the sudden or impulsive photoactivation of the wing-flapping mode is triggered by a density depletion between the ethylenic bonds and density accumulation between the π_CC_ bonds (Figure 7b). The change of the bonding energy between the atoms launches nuclear motions in which the distance between the ethylenic halves *d* decreases and the distance between the ethylenic bonds increases. From Figure 7a, it is also noted that the stretching mode between the ethylenic C atoms is significantly coupled to the electronic transition.

The wing-flapping mode represents a good approximation to the barrierless coordinate driving the deactivation from FC-S_2_**NBD** to the S_2_/S_1_ and S_1_/S_0_ MECIs. Experiments have also shown that this mode plays an important role in **NBD** deactivation.^49^ The wing-flapping vibrational period, computed with SA6-CASSCF(4,7)/ANO-S-VDZP, is 75 fs, in very good agreement with the experiments (80 fs).^58^ Thus, deactivation from S_2_ occurs within the first vibrational period supporting the conclusion that the wing-flapping mode must be impulsively activated upon photon absorption.

**Figure 8:**
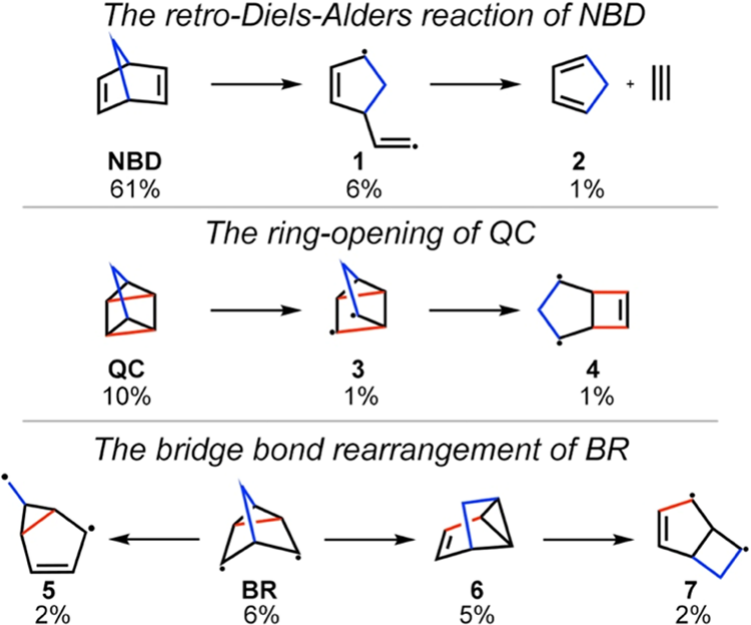
Side reactions observed in the gas-phase **NBD** trajectories with the ratios of products or intermediates. The methylene bridge and the newly formed σ_CC_-bonds in **QC** are highlighted in blue and red, respectively.

Besides the major products, our photodynamics simulations also identified several side reaction rearrangement pathways (Figure 8). These reactions feature breaking σ_CC_-bonds; CASSCF rotates the σ_CC_-orbitals into the (4,7) active space as the σ_CC_-bonds stretch beyond their equilibrium geometries during the trajectories. The QYs for the ring-opening and rearrangement pathways are relatively minor (2–6%) compared to the reversion to **NBD** (61%) and the [2 + 2]-cycloaddition pathways (10%). The predicted side reactions suggest a qualitative agreement with the photodegradation of substituted **NBDs** observed in experiments when the excitation localized on the **NBD** unit is probed.^88^

**Figure 9:**
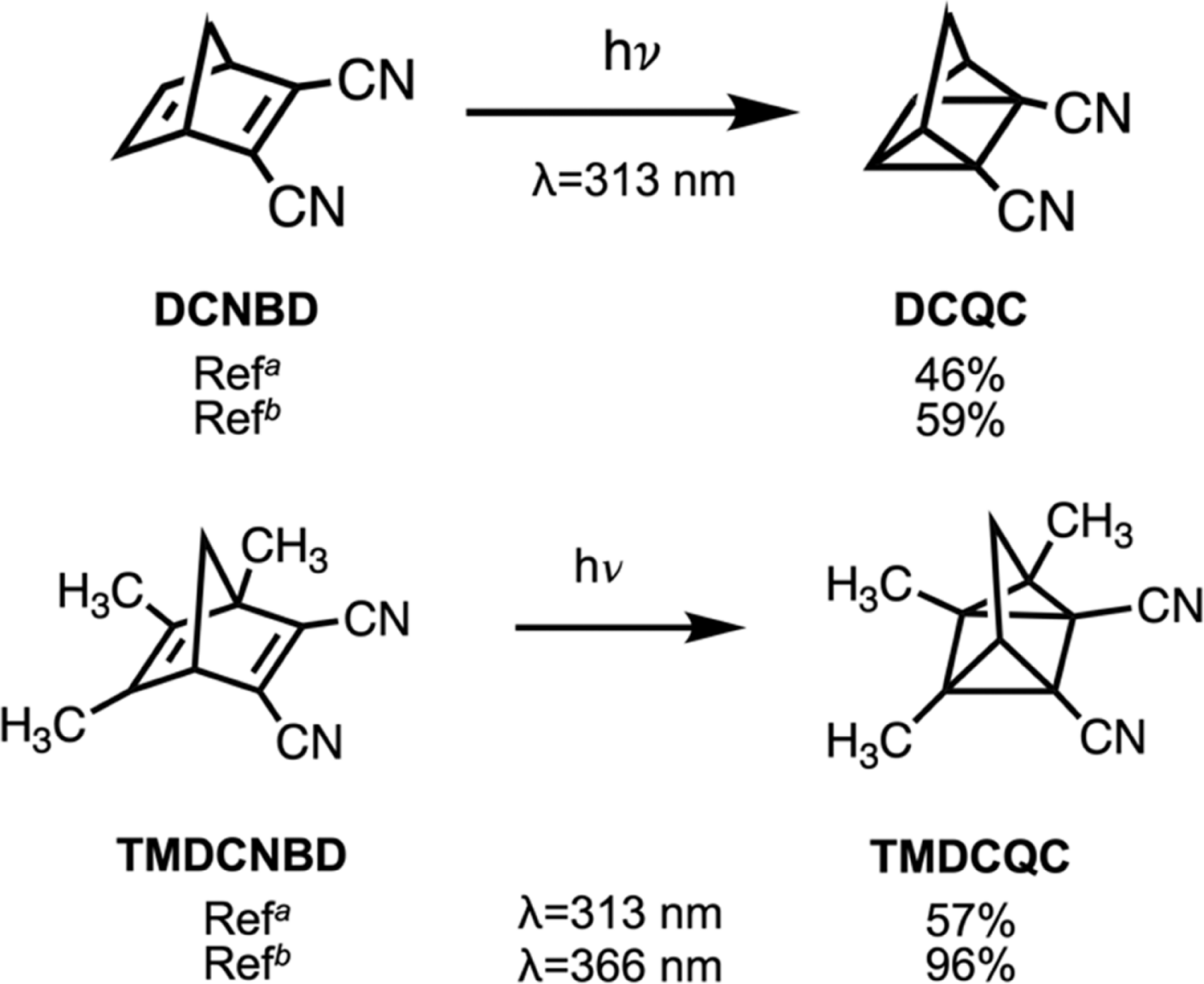
Literature reported [2 + 2]-cycloaddition of the cyano-substituted **NBD** systems at different wavelengths. ^*a*^Ref (63). ^*b*^Ref (64).

We continue to explore the substituent effects on **NBD** that might change the photophysical properties and block the competing cycloreversions and rearrangements. The literature reports that the cyano groups can red-shift the absorption wavelength of **NBD** to 334–366 nm with enhanced **QC** yields of 57–96% (Figure 9).^63,64^ A recent work also investigated the spectroscopic properties of cyano-substituted **NBD**.^89^

For simplicity, we generated the structure of **DMDCNBD** by replacing the methyl group with H on the bridge-head carbon based on the analogous **TMDCNBD** in Figure 9. The dimethyl and dicyano groups on each side provide donor–acceptor-type electronic inductions. Table 2 collects the computed vertical excitation energies and electronic transitions for **DMDCNBD**.

**Table 2:** Vertical Excitation Energies with the Principal Electronic Configurations of **DMDCNBD** Calculated with the Relaxed Geometry in the Ground State

	S_1_		S_2_		S_3_	
ωB97XD/aug-cc-pVDZ	4.03	π_4_ → π_5_*	5.16	π_3_ → π_5_*	5.88	π_2_ → π_5_*
SA6-CASSCF(8,6)/ANO-S-VDZP	5.68	π_4_ → π_5_*	7.30	π_2_ → π_5_*	7.40	π_3_ → π_5_*
						π_4_→ π_5_*
XMS(6)-CASPT2(8,6)/ANO-S-VDZP	4.33	π_4_ → π_5_*	5.70	π_3_ → π_5_*	6.70	π_2_ → π_5_*

The TD-ωB97XD/aug-cc-pVDZ calculation shows the S_1_ energy of 4.03 eV, which is comparable to the experimental value reported for its analogue **TMDCNBD** (3.71 eV).^88^ The S_1_ and S_2_ are described by π–π* excitations between the π_CC_-bonds; the S_3_ excites one electron from the π_CN_ to the π_CC_*-orbital. The SA6-CASSCF(8,6)/ANO-S-VDZP calculations overestimate the excitation energies compared to the XMS(6)-CASPT2(8,6)/ANO-S-VDZP results. Both methods predict the same π–π* excitation for S_1_. However, the π_CN_ excitation is underestimated to S_2_ by SA6-CASSCF(8,6)/ANO-S-VDZP and S_3_ becomes a doubly excited state. The S_2_ and S_3_ description by XMS(6)-CASPT2(8,6)/ANO-S-VDZP results agree with TD-ωB97XD/aug-cc-pVDZ. Together, the vertical excitation calculations show that the substitution with cyano groups substantially lowers the excitation energy and eliminates the contribution of Rydberg excitations. Considering the large S_1_/S_2_ state energy gap, the SA6-CASSCF(8,6)/ANO-S-VDZP method is reasonable for studying the S_1_ → S_0_ decay and subsequent photochemical reaction pathways.

**Figure 10:**
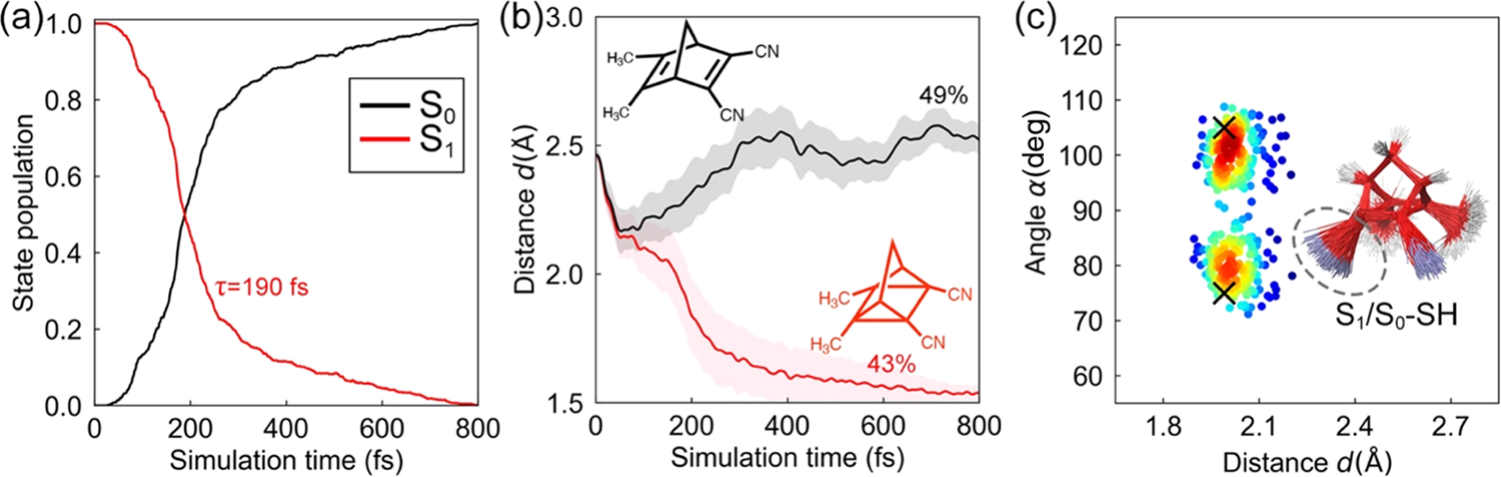
(a) State population, (b) average π_CC_-distance, and (c) surface hopping distributions of 492 **DMDCNBD** trajectories in the gas phase. In panel (b), the shaded regions render a half standard deviation to the average values to show the diversity of the trajectories. In panel (c), the “X” denotes the positions of the S_1_/S_0_ MECIs. The colors from blue to red represent the accumulation of the surface hopping points from low to high, evaluated by Gaussian kernel density estimation. Overlays of the 200 randomly selected S_1_/S_0_ surface hopping structures are shown inside panel (c). The gray circle highlights the pyramidalization of the π_CC_ bond.

The vertical excitations to the FC regions of **DMDCNBD** present two separate absorptions without Rydberg character (Figure S5). The first peak corresponds to the S_1_ excitation, and the second peak covers the excitation to S_2_–S_4_. As such, our photodynamics simulations for **DMDCNBD** start at S_1_ assuming initial excitations via a feasible light source (e.g., 254 nm, 4.8 eV). The simulation time is 800 fs. We collected 492 trajectories to obtain statistically converged results (Figures 10 and S6).

In 800 fs, 98% of the trajectories finished in the S_0_ state and 2% in S_1_. Figure 9a plots the S_1_ population dynamics with a time constant τ = 190 fs. This time constant is longer than the values for **NBD**, suggesting that the substituent effects slow the S_1_ → S_0_ decay. This finding agrees with a recent study on the ultrafast dynamics of substituted **NBD**s that shows that the excited-state lifetimes are notably increased.^87^Figure 9b illustrates the trajectories toward **DMDCQC** and back to **DMDCNBD**. The bifurcation takes place when the π_CC_-distances are longer than 2 Å, where the rhomboidal angle distribute within 85–95° (Figure S6a). The **DMDCQC** forms immediately as the π_CC_-bond length reduces. The predicted yields **DMDCNBD** and **DMDCQC** are 49 and 43%, respectively, resulting in a 1:1 ratio of **QC**/**NBD**. The **DMDCQC** yield is comparable to the reported one for its analogue **TMDCQC**, 57% at 366 nm (Figure 9). These findings indicate the substituent groups facilitate the [2 + 2]-cycloaddition. Moreover, the singlet biradical intermediate observed in NBD decreases to 1% and the overall side products decrease to 9% (Figure S7). These results support our argument that the substituent groups block the cycloreversions and rearrangements.

Figure 9c shows a scatter plot for the geometrical distributions of the S_1_/S_0_ hopping point structures. The S_1_/S_0_ surface hopping display symmetric angular distribution at *α* = 80 and 102° with *d* = 2.00 Å. The average π_CC_ distance at the S_1_/S_0_ hopping points is shorter than that at the trajectory bifurcation (>2 Å). This result suggests the substituent effects favor the [2 + 2]-cycloaddition, where the trajectories tend to form **DMDCQC** even before approaching the S_1_/S_0_ crossing seam around the S_1_/S_0_-MECI at *d* = 1.99 Å and *α* = 75° (105°). The overlay of the S_1_/S_0_ hopping points highlights the pyramidalized π_CC_ bond that we observed in the unsubstituted **NBD**. Collectively, our photodynamics simulations of **NBD** show the substituent effects that considerably increase the chemoselectivity of [2 + 2]-cycloaddition raising the QY of **QC**.

## 4 Conclusions

We have performed photodynamics simulations with CASSCF calculations to elucidate the deactivation mechanisms of photoexcited **NBD**. The vertical excitation energies reveal the significant role of Rydberg states in the absorption of unsubstituted **NBD**, in line with previous experimental and computational studies. Substitution with methyl and cyano groups eliminate the Rydberg excitation resulting in dominant ππ* configurations. We propagated 577 trajectories of **NBD** from the bright S_2_ state in 500 fs, effectively connecting the FC region to the product region, which identified products beyond **QC**. Besides forming the rhomboidal structures at the S_1_/S_0_ surface hopping points, we also observed the pyramidalization of the π_CC_ bonds, promoting the nonradiative decay. The predicted QYs of **NBD**, **QC**, and **BR** are 61, 10, and 6%, respectively. We performed photodynamics simulations for another 492 trajectories for **DMDCNBD** to reveal the substituent effects on the photochemical reaction mechanisms of **NBD**. The trajectories show that **DMDCNBD** undergoes the same mechanism as **NBD**, forming a rhomboidal structure with pyramidalized π_CC_ bonds at the S_1_/S_0_ crossing seam. However, the trajectories of **DMDCNBD** bifurcate faster than those of the unsubstituted **NBD**, showing strong preference for [2 + 2]-cycloaddition reactions. The predicted QYs of **DMDCNBD** and **DMDCQC** are 49 and 43%, respectively, indicating that the substituent effects increase the QY of **QC**. Moreover, the overall yields of the side products are considerably reduced, supporting our conclusion that the substituent effects can block competing pathways.

## Data Availability

The data underlying this study is available in the published article and its Supporting Information.

## Supporting Information Available

Cartesian coordinates of optimized structures, electronic energies, and additional information on trajectories (PDF)

## References

[ref1] Carey G. H.; Abdelhady A. L.; Ning Z.; Thon S. M.; Bakr O. M.; Sargent E. H. Colloidal Quantum Dot Solar Cells. Chem. Rev. 2015, 115, 12732–12763. 10.1021/acs.chemrev.5b00063. 26106908

[ref2] Lu L.; Zheng T.; Wu Q.; Schneider A. M.; Zhao D.; Yu L. Recent Advances in Bulk Heterojunction Polymer Solar Cells. Chem. Rev. 2015, 115, 12666–12731. 10.1021/acs.chemrev.5b00098. 26252903

[ref3] Kim J. Y.; Lee J.-W.; Jung H. S.; Shin H.; Park N.-G. High-Efficiency Perovskite Solar Cells. Chem. Rev. 2020, 120, 7867–7918. 10.1021/acs.chemrev.0c00107. 32786671

[ref4] Peng W.; Rupich S. M.; Shafiq N.; Gartstein Y. N.; Malko A. V.; Chabal Y. J. Silicon Surface Modification and Characterization for Emergent Photovoltaic Applications Based on Energy Transfer. Chem. Rev. 2015, 115, 12764–12796. 10.1021/acs.chemrev.5b00085. 26244614

[ref5] Nayak P. K.; Mahesh S.; Snaith H. J.; Cahen D. Photovoltaic solar cell technologies: analysing the state of the art. Nat. Rev. Mater. 2019, 4, 269–285. 10.1038/s41578-019-0097-0.

[ref6] Cox J. M.; Mileson B.; Sadagopan A.; Lopez S. A. Molecular Recognition and Band Alignment in 3D Covalent Organic Frameworks for Cocrystalline Organic Photovoltaics. J. Phys. Chem. C 2020, 124, 9126–9133. 10.1021/acs.jpcc.0c00087.

[ref7] Wang W. H.; Himeda Y.; Muckerman J. T.; Manbeck G. F.; Fujita E. CO2 Hydrogenation to Formate and Methanol as an Alternative to Photo- and Electrochemical CO2 Reduction. Chem. Rev. 2015, 115, 12936–12973. 10.1021/acs.chemrev.5b00197. 26335851

[ref8] White J. L.; Baruch M. F.; Pander J. E. III; Hu Y.; Fortmeyer I. C.; Park J. E.; Zhang T.; Liao K.; Gu J.; Yan Y.; Shaw T. W.; Abelev E.; Bocarsly A. B. Light-Driven Heterogeneous Reduction of Carbon Dioxide: Photocatalysts and Photoelectrodes. Chem. Rev. 2015, 115, 12888–12935. 10.1021/acs.chemrev.5b00370. 26444652

[ref9] Morikawa T.; Sato S.; Sekizawa K.; Suzuki T. M.; Arai T. Solar-Driven CO2 Reduction Using a Semiconductor/Molecule Hybrid Photosystem: From Photocatalysts to a Monolithic Artificial Leaf. Acc. Chem. Res. 2022, 55, 933–943. 10.1021/acs.accounts.1c00564. 34851099

[ref10] Nakada A.; Kumagai H.; Robert M.; Ishitani O.; Maeda K. Molecule/Semiconductor Hybrid Materials for Visible-Light CO2 Reduction: Design Principles and Interfacial Engineering. Acc. Mater. Res. 2021, 2, 458–470. 10.1021/accountsmr.1c00060.

[ref11] Kang D.; Kim T. W.; Kubota S. R.; Cardiel A. C.; Cha H. G.; Choi K. S. Electrochemical Synthesis of Photoelectrodes and Catalysts for Use in Solar Water Splitting. Chem. Rev. 2015, 115, 12839–12887. 10.1021/acs.chemrev.5b00498. 26538328

[ref12] Wang Q.; Domen K. Particulate Photocatalysts for Light-Driven Water Splitting: Mechanisms, Challenges, and Design Strategies. Chem. Rev. 2020, 120, 919–985. 10.1021/acs.chemrev.9b00201. 31393702

[ref13] Liu Y.; Chen Y.; Ma S.; Liu X.; Zhang X.; Zou J.-J.; Pan L. Synthesis of advanced fuel with density higher than 1 g/mL by photoinduced [2 + 2] cycloaddition of norbornene. Fuel 2022, 318, 123629 10.1016/j.fuel.2022.123629.

[ref14] Xie J.; Zhang X.; Shi C.; Pan L.; Hou F.; Nie G.; Xie J.; Liu Q.; Zou J.-J. Self-photosensitized [2 + 2] cycloaddition for synthesis of high-energy-density fuels. Sustainable Energy Fuels 2020, 4, 911–920. 10.1039/c9se00863b.

[ref15] Calbo J.; Weston C. E.; White A. J.; Rzepa H. S.; Contreras-Garcia J.; Fuchter M. J. Tuning Azoheteroarene Photoswitch Performance through Heteroaryl Design. J. Am. Chem. Soc. 2017, 139, 1261–1274. 10.1021/jacs.6b11626. 28009517

[ref16] Gonzalez A.; Kengmana E. S.; Fonseca M. V.; Han G. G. D. Solid-state photoswitching molecules: structural design for isomerization in condensed phase. Mater. Today Adv. 2020, 6, 100058 10.1016/j.mtadv.2020.100058.

[ref17] Sun C. L.; Wang C.; Boulatov R. Applications of Photoswitches in the Storage of Solar Energy. ChemPhotoChem 2019, 3, 268–283. 10.1002/cptc.201900030.

[ref18] Saydjari A. K.; Weis P.; Wu S. Spanning the Solar Spectrum: Azopolymer Solar Thermal Fuels for Simultaneous UV and Visible Light Storage. Adv. Energy Mater. 2017, 7, 1601622 10.1002/aenm.201601622.

[ref19] Xu X.; Wang G. Molecular Solar Thermal Systems towards Phase Change and Visible Light Photon Energy Storage. Small 2022, 18, 2107473 10.1002/smll.202107473. 35132792

[ref20] Wang Z.; Erhart P.; Li T.; Zhang Z.-Y.; Sampedro D.; Hu Z.; Wegner H. A.; Brummel O.; Libuda J.; Nielsen M. B.; Moth-Poulsen K. Storing energy with molecular photoisomers. Joule 2021, 5, 3116–3136. 10.1016/j.joule.2021.11.001.

[ref21] Lennartson A.; Roffey A.; Moth-Poulsen K. Designing photoswitches for molecular solar thermal energy storage. Tetrahedron Lett. 2015, 56, 1457–1465. 10.1016/j.tetlet.2015.01.187.

[ref22] Kolpak A. M.; Grossman J. C. Azobenzene-functionalized carbon nanotubes as high-energy density solar thermal fuels. Nano Lett. 2011, 11, 3156–3162. 10.1021/nl201357n. 21688811

[ref23] Wang Z.; Losantos R.; Sampedro D.; Morikawa M.-a.; Börjesson K.; Kimizuka N.; Moth-Poulsen K. Demonstration of an azobenzene derivative based solar thermal energy storage system. J. Mater. Chem. A 2019, 7, 15042–15047. 10.1039/c9ta04905c.

[ref24] Wu S.; Butt H. J. Solar-Thermal Energy Conversion and Storage Using Photoresponsive Azobenzene-Containing Polymers. Macromol. Rapid Commun. 2020, 41, 1900413 10.1002/marc.201900413. 31737964

[ref25] Jones G.; Reinhardt T. E.; Bergmark W. R. Photon energy storage in organic materials— The case of linked anthracenes. Sol. Energy 1978, 20, 241–248. 10.1016/0038-092x(78)90103-2.

[ref26] Ganguly G.; Sultana M.; Paul A. Designing Efficient Solar-Thermal Fuels with [n.n](9,10)Anthracene Cyclophanes: A Theoretical Perspective. J. Phys. Chem. Lett. 2018, 9, 328–334. 10.1021/acs.jpclett.7b03170. 29256618

[ref27] Börjesson K.; Lennartson A.; Moth-Poulsen K. Fluorinated fulvalene ruthenium compound for molecular solar thermal applications. J. Fluorine Chem. 2014, 161, 24–28. 10.1016/j.jfluchem.2014.01.012.

[ref28] Boese R.; Cammack J. K.; Matzger A. J.; Pflug K.; Tolman W. B.; Vollhardt K. P. C.; Weidman T. W. Photochemistry of (Fulvalene)tetracarbonyldiruthenium and Its Derivatives: Efficient Light Energy Storage Devices. J. Am. Chem. Soc. 1997, 119, 6757–6773. 10.1021/ja9707062.

[ref29] Moth-Poulsen K.; Ćoso D.; Börjesson K.; Vinokurov N.; Meier S. K.; Majumdar A.; Vollhardt K. P. C.; Segalman R. A. Molecular solar thermal (MOST) energy storage and release system. Energy Environ. Sci. 2012, 5, 8534 10.1039/c2ee22426g.

[ref30] Börjesson K.; Coso D.; Gray V.; Grossman J. C.; Guan J.; Harris C. B.; Hertkorn N.; Hou Z.; Kanai Y.; Lee D.; Lomont J. P.; Majumdar A.; Meier S. K.; Moth-Poulsen K.; Myrabo R. L.; Nguyen S. C.; Segalman R. A.; Srinivasan V.; Tolman W. B.; Vinokurov N.; Vollhardt K. P. C.; Weidman T. W. Exploring the potential of fulvalene dimetals as platforms for molecular solar thermal energy storage: computations, syntheses, structures, kinetics, and catalysis. Chem.—Eur. J. 2014, 20, 15587–15604. 10.1002/chem.201404170. 25284044

[ref31] Vlasceanu A.; Koerstz M.; Skov A. B.; Mikkelsen K. V.; Nielsen M. B. Multistate Photoswitches: Macrocyclic Dihydroazulene/Azobenzene Conjugates. Angew. Chem. 2018, 130, 6177–6180. 10.1002/ange.201712942. 29566296

[ref32] Skov A. B.; Petersen J. F.; Elm J.; Frandsen B. N.; Santella M.; Kilde M. D.; Kjaergaard H. G.; Mikkelsen K. V.; Nielsen M. B. Towards Storage of Solar Energy in Photochromic Molecules: Benzannulation of the Dihydroazulene/Vinylheptafulvene Couple. ChemPhotoChem 2017, 1, 206–212. 10.1002/cptc.201600046.

[ref33] Mogensen J.; Christensen O.; Kilde M. D.; Abildgaard M.; Metz L.; Kadziola A.; Jevric M.; Mikkelsen K. V.; Nielsen M. B. Molecular Solar Thermal Energy Storage Systems with Long Discharge Times Based on the Dihydroazulene/Vinylheptafulvene Couple. Eur. J. Org. Chem. 2019, 2019, 1986–1993. 10.1002/ejoc.201801776.

[ref34] Kilde M. D.; Arroyo P. G.; Gertsen A. S.; Mikkelsen K. V.; Nielsen M. B. Molecular solar thermal systems - control of light harvesting and energy storage by protonation/deprotonation. RSC Adv. 2018, 8, 6356–6364. 10.1039/c7ra13762a. 35540374 PMC9078237

[ref35] Hillers-Bendtsen A. E.; Quant M.; Moth-Poulsen K.; Mikkelsen K. V. Investigation of the Structural and Thermochemical Properties of [2.2.2]-Bicyclooctadiene Photoswitches. J. Phys. Chem. A 2021, 125, 10330–10339. 10.1021/acs.jpca.1c07737. 34809434

[ref36] Quant M.; Hillers-Bendtsen A. E.; Ghasemi S.; Erdelyi M.; Wang Z.; Muhammad L. M.; Kann N.; Mikkelsen K. V.; Moth-Poulsen K. Synthesis, characterization and computational evaluation of bicyclooctadienes towards molecular solar thermal energy storage. Chem. Sci. 2022, 13, 834–841. 10.1039/d1sc05791j. 35173948 PMC8768882

[ref37] Mansø M.; Tebikachew B. E.; Moth-Poulsen K.; Nielsen M. B. Heteroaryl-linked norbornadiene dimers with redshifted absorptions. Org. Biomol. Chem. 2018, 16, 5585–5590. 10.1039/c8ob01470a. 30051895

[ref38] Mansø M.; Fernandez L.; Wang Z.; Moth-Poulsen K.; Nielsen M. B. Donor-Acceptor Substituted Benzo-, Naphtho- and Phenanthro-Fused Norbornadienes. Molecules 2020, 25, 322 10.3390/molecules25020322. 31941131 PMC7024342

[ref39] Jevric M.; Petersen A. U.; Manso M.; Kumar Singh S.; Wang Z.; Dreos A.; Sumby C.; Nielsen M. B.; Borjesson K.; Erhart P.; Moth-Poulsen K. Norbornadiene-Based Photoswitches with Exceptional Combination of Solar Spectrum Match and Long-Term Energy Storage. Chem.—Eur. J. 2018, 24, 12767–12772. 10.1002/chem.201802932. 29978927

[ref40] Wang Z.; Roffey A.; Losantos R.; Lennartson A.; Jevric M.; Petersen A. U.; Quant M.; Dreos A.; Wen X.; Sampedro D.; Borjesson K.; Moth-Poulsen K. Macroscopic heat release in a molecular solar thermal energy storage system. Energy Environ. Sci. 2019, 12, 187–193. 10.1039/c8ee01011k.

[ref41] Orrego-Hernández J.; Dreos A.; Moth-Poulsen K. Engineering of Norbornadiene/Quadricyclane Photoswitches for Molecular Solar Thermal Energy Storage Applications. Acc. Chem. Res. 2020, 53, 1478–1487. 10.1021/acs.accounts.0c00235. 32662627 PMC7467572

[ref42] Dubonosov A. D.; Bren V. A.; Chernoivanov V. A. Norbornadiene–quadricyclane as an abiotic system for the storage of solar energy. Russ. Chem. Rev. 2002, 71, 917–927. 10.1070/RC2002v071n11ABEH000745.

[ref43] Nucci M.; Marazzi M.; Frutos L. M. Mechanochemical Improvement of Norbornadiene-Based Molecular Solar–Thermal Systems Performance. ACS Sustainable Chem. Eng. 2019, 7, 19496–19504. 10.1021/acssuschemeng.9b04503.

[ref44] Dauben W. G.; Cargill R. L. Photochemical transformations—VIII. Tetrahedron 1961, 15, 197–201. 10.1016/0040-4020(61)80026-4.

[ref45] Cristol S. J.; Snell R. L. Bridged Polycyclic Compounds. VI. The Photoisomerization of Bicyclo [2,2,1]hepta-2,5-diene-2,3-dicarboxylic Acid to Quadricyclo [2,2,1,02,6,03,5]heptane-2,3-dicarboxylic Acid1,2. J. Am. Chem. Soc. 1958, 80, 1950–1952. 10.1021/ja01541a043.

[ref46] Frey H. M. 63. The thermal isomerisation of quadricyclene (quadricyclo-[2,2,1,02,6,03,5]heptane). Part I. The gas-phase reaction. J. Chem. Soc. 1964, 365–367. 10.1039/jr9640000365.

[ref47] Hammond G. S.; Turro N. J.; Fischer A. Photosensitized Cycloaddition Reactions1. J. Am. Chem. Soc. 1961, 83, 4674–4675. 10.1021/ja01483a051.

[ref48] Dilling W. L. Intramolecular Photochemical Cycloaddition of Nonconjugated Olefins. Chem. Rev. 1966, 66, 373–393. 10.1021/cr60242a002.

[ref49] Fuß W.; Pushpa K. K.; Schmid W. E.; Trushin S. A. Ultrafast [2 + 2]-cycloaddition in norbornadiene. Photochem. Photobiol. Sci. 2002, 1, 60–66. 10.1039/b107442c. 12659150

[ref50] Miki S.; Asako Y.; Yoshida Z.-i. Photochromic Solid Films Prepared by Doping with Donor–Acceptor Norbornadienes. Chem. Lett. 1987, 16, 195–198. 10.1246/cl.1987.195.

[ref51] Dreos A.; Wang Z.; Udmark J.; Ström A.; Erhart P.; Börjesson K.; Nielsen M. B.; Moth-Poulsen K. Liquid Norbornadiene Photoswitches for Solar Energy Storage. Adv. Energy Mater. 2018, 8, 1703401 10.1002/aenm.201703401.

[ref52] Quant M.; Lennartson A.; Dreos A.; Kuisma M.; Erhart P.; Borjesson K.; Moth-Poulsen K. Low Molecular Weight Norbornadiene Derivatives for Molecular Solar-Thermal Energy Storage. Chem.—Eur. J. 2016, 22, 13265–13274. 10.1002/chem.201602530. 27492997 PMC5096010

[ref53] Mansø M.; Kilde M. D.; Singh S. K.; Erhart P.; Moth-Poulsen K.; Nielsen M. B. Dithiafulvene derivatized donor-acceptor norbornadienes with redshifted absorption. Phys. Chem. Chem. Phys. 2019, 21, 3092–3097. 10.1039/c8cp07744d. 30672939

[ref54] Gray V.; Lennartson A.; Ratanalert P.; Borjesson K.; Moth-Poulsen K. Diaryl-substituted norbornadienes with red-shifted absorption for molecular solar thermal energy storage. Chem. Commun. 2014, 50, 5330–5332. 10.1039/c3cc47517d. 24280803

[ref55] Elholm J. L.; Hillers-Bendtsen A. E.; Hölzel H.; Moth-Poulsen K.; Mikkelsen K. V. High Throughput Screening of Norbornadiene/Quadricyclane Derivates for Molecular Solar Thermal Energy Storage. Phys. Chem. Chem. Phys. 2022, 24, 28956–28964. 10.1039/d2cp03032b. 36416497

[ref56] Ree N.; Koerstz M.; Mikkelsen K. V.; Jensen J. H. Virtual screening of norbornadiene-based molecular solar thermal energy storage systems using a genetic algorithm. J. Chem. Phys. 2021, 155, 184105 10.1063/5.0063694. 34773961

[ref57] Roos B. O.; Merchan M.; McDiarmid R.; Xing X. Theoretical and Experimental Determination of the Electronic Spectrum of Norbornadiene. J. Am. Chem. Soc. 1994, 116, 5927–5936. 10.1021/ja00092a049.

[ref58] Xing X.; Gedanken A.; Sheybani A.-H.; McDiarmid R. The 198-225-nm Transition of Norbornadiene. J. Phys. Chem. A 1994, 98, 8302–8309. 10.1021/j100085a007.

[ref59] Antol I. Photodeactivation paths in norbornadiene. J. Comput. Chem. 2013, 34, 1439–1445. 10.1002/jcc.23270. 23553256

[ref60] Valentini A.; van den Wildenberg S.; Remacle F. Selective bond formation triggered by short optical pulses: quantum dynamics of a four-center ring closure. Phys. Chem. Chem. Phys. 2020, 22, 22302–22313. 10.1039/d0cp03435e. 33006338

[ref61] Jorner K.; Dreos A.; Emanuelsson R.; El Bakouri O.; Fdez; Galván I.; Börjesson K.; Feixas F.; Lindh R.; Zietz B.; Moth-Poulsen K.; Ottosson H. Unraveling factors leading to efficient norbornadiene–quadricyclane molecular solar-thermal energy storage systems. J. Mater. Chem. A 2017, 5, 12369–12378. 10.1039/c7ta04259k.

[ref62] Kuisma M. J.; Lundin A. M.; Moth-Poulsen K.; Hyldgaard P.; Erhart P. Comparative Ab-Initio Study of Substituted Norbornadiene-Quadricyclane Compounds for Solar Thermal Storage. J. Phys. Chem. C 2016, 120, 3635–3645. 10.1021/acs.jpcc.5b11489. PMC4780837 26966476

[ref63] Harel Y.; Adamson A. W.; Kutal C.; Grutsch P. A.; Yasufuku K. Photocalorimetry. 6. Enthalpies of isomerization of norbornadiene and of substituted norbornadienes to corresponding quadricyclenes. J. Phys. Chem. A 1987, 91, 901–904. 10.1021/j100288a027.

[ref64] Yoshida Z.-i. New molecular energy storage systems. J. Photochem. 1985, 29, 27–40. 10.1016/0047-2670(85)87059-3.

[ref65] Coppola F.; Nucci M.; Marazzi M.; Rocca D.; Pastore M. Norbornadiene/Quadricyclane System in the Spotlight: The Role of Rydberg States and Dynamic Electronic Correlation in a Solar-Thermal Building Block. ChemPhotoChem 2023, e202200214 10.1002/cptc.202200214.

[ref66] Chai J. D.; Head-Gordon M. Systematic optimization of long-range corrected hybrid density functionals. J. Chem. Phys. 2008, 128, 084106 10.1063/1.2834918. 18315032

[ref67] Stanton J. F.; Bartlett R. J. The equation of motion coupled-cluster method. A systematic biorthogonal approach to molecular excitation energies, transition probabilities, and excited state properties. J. Chem. Phys. 1993, 98, 7029–7039. 10.1063/1.464746.

[ref68] Kendall R. A.; Dunning T. H.; Harrison R. J. Electron affinities of the first-row atoms revisited. Systematic basis sets and wave functions. J. Chem. Phys. 1992, 96, 6796–6806. 10.1063/1.462569.

[ref69] Pierloot K.; Dumez B.; Widmark P.-O.; Roos B. r. O. Density matrix averaged atomic natural orbital (ANO) basis sets for correlated molecular wave functions. Theor. Chim. Acta 1995, 90, 87–114. 10.1007/bf01113842.

[ref70] Pou-Amérigo R.; Merchán M.; Nebot-Gil I.; Widmark P.-O.; Roos B. O. Density matrix averaged atomic natural orbital (ANO) basis sets for correlated molecular wave functions. Theor. Chim. Acta 1995, 92, 149–181. 10.1007/bf01114922.

[ref71] Widmark P.-O.; Malmqvist P.-k.; Roos B. r. O. Density matrix averaged atomic natural orbital (ANO) basis sets for correlated molecular wave functions. Theor. Chim. Acta 1990, 77, 291–306. 10.1007/bf01120130.

[ref72] Widmark P.-O.; Persson B. J.; Roos B. r. O. Density matrix averaged atomic natural orbital (ANO) basis sets for correlated molecular wave functions. Theor. Chim. Acta 1991, 79, 419–432. 10.1007/bf01112569.

[ref73] Plasser F.; Mewes S. A.; Dreuw A.; Gonzalez L. Detailed Wave Function Analysis for Multireference Methods: Implementation in the Molcas Program Package and Applications to Tetracene. J. Chem. Theory Comput. 2017, 13, 5343–5353. 10.1021/acs.jctc.7b00718. 28972759

[ref74] Fdez Galván I.; Vacher M.; Alavi A.; Angeli C.; Aquilante F.; Autschbach J.; Bao J. J.; Bokarev S. I.; Bogdanov N. A.; Carlson R. K.; Chibotaru L. F.; Creutzberg J.; Dattani N.; Delcey M. G.; Dong S. S.; Dreuw A.; Freitag L.; Frutos L. M.; Gagliardi L.; Gendrón F.; Giussani A.; González L.; Grell G.; Guo M.; Hoyer C. E.; Johansson M.; Keller S.; Knecht S.; Kovacevic G.; Kallman E.; Li Manni G.; Lundberg M.; Ma Y.; Mai S.; Malhado J. P.; Malmqvist P. A.; Marquetand P.; Mewes S. A.; Norell J.; Olivucci M.; Oppel M.; Phung Q. M.; Pierloot K.; Plasser F.; Reiher M.; Sand A. M.; Schapiro I.; Sharma P.; Stein C. J.; Sørensen L. K.; Truhlar D. G.; Ugandi M.; Ungur L.; Valentini A.; Vancoillie S.; Veryazov V.; Weser O.; Wesołowski T. A.; Widmark P-O.; Wouters S.; Zech A.; Zobel J. P.; Lindh R. OpenMolcas: From Source Code to Insight. J. Chem. Theory Comput. 2019, 15, 5925–5964. 10.1021/acs.jctc.9b00532. 31509407

[ref75] Neese F. The ORCA program system. Wiley Interdiscip. Rev.: Comput. Mol. Sci. 2012, 2, 73–78. 10.1002/wcms.81.

[ref76] Crespo-Otero R.; Barbatti M. Recent Advances and Perspectives on Nonadiabatic Mixed Quantum-Classical Dynamics. Chem. Rev. 2018, 118, 7026–7068. 10.1021/acs.chemrev.7b00577. 29767966

[ref77] Tully J. C. Molecular dynamics with electronic transitions. J. Chem. Phys. 1990, 93, 1061–1071. 10.1063/1.459170.

[ref78] Pittner J.; Lischka H.; Barbatti M. Optimization of mixed quantum-classical dynamics: Time-derivative coupling terms and selected couplings. Chem. Phys. 2009, 356, 147–152. 10.1016/j.chemphys.2008.10.013.

[ref79] Granucci G.; Persico M. Critical appraisal of the fewest switches algorithm for surface hopping. J. Chem. Phys. 2007, 126, 134114 10.1063/1.2715585. 17430023

[ref80] Crespo-Otero R.; Barbatti M. Spectrum simulation and decomposition with nuclear ensemble: formal derivation and application to benzene, furan and 2-phenylfuran. Theor. Chem. Acc. 2012, 131, 1237 10.1007/s00214-012-1237-4.

[ref81] Dahl J. P.; Springborg M. The Morse oscillator in position space, momentum space, and phase space. J. Chem. Phys. 1988, 88, 4535–4547. 10.1063/1.453761.

[ref82] Li J.; Reiser P.; Boswell B. R.; Eberhard A.; Burns N. Z.; Friederich P.; Lopez S. A. Automatic discovery of photoisomerization mechanisms with nanosecond machine learning photodynamics simulations. Chem. Sci. 2021, 12, 5302–5314. 10.1039/d0sc05610c. 34163763 PMC8179587

[ref83] Li J.; Lopez S. A. Excited-State Distortions Promote the Photochemical 4pi-Electrocyclizations of Fluorobenzenes via Machine Learning Accelerated Photodynamics Simulations. Chem.—Eur. J. 2022, 28, e202200651 10.1002/chem.202200651. 35474348

[ref84] Ben-Nun M.; Martínez T. J. Photodynamics of ethylene: ab initio studies of conical intersections. Chem. Phys. 2000, 259, 237–248. 10.1016/s0301-0104(00)00194-4.

[ref85] Hernández F. J.; Bonafe F. P.; Aradi B.; Frauenheim T.; Sanchez C. G. Simulation of Impulsive Vibrational Spectroscopy. J. Phys. Chem. A 2019, 123, 2065–2072. 10.1021/acs.jpca.9b00307. 30767532

[ref86] Bonafé F. P.; Hernandez F. J.; Aradi B.; Frauenheim T.; Sanchez C. G. Fully Atomistic Real-Time Simulations of Transient Absorption Spectroscopy. J. Phys. Chem. Lett. 2018, 9, 4355–4359. 10.1021/acs.jpclett.8b01659. 30024765

[ref87] Cerezo J.; Santoro F. FCclasses3: Vibrationally-resolved spectra simulated at the edge of the harmonic approximation. J. Comput. Chem. 2023, 44, 626–643. 10.1002/jcc.27027. 36380723 PMC10100349

[ref88] Alex W.; Lorenz P.; Henkel C.; Clark T.; Hirsch A.; Guldi D. M. Solar Energy Storage: Competition between Delocalized Charge Transfer and Localized Excited States in the Norbornadiene to Quadricyclane Photoisomerization. J. Am. Chem. Soc. 2022, 144, 153–162. 10.1021/jacs.1c04322. 34958548

[ref89] Martin-Drumel M.-A.; Spaniol J.-T.; Hölzel H.; Agundez M.; Cernicharo J.; Moth-Poulsen K.; Jacovella U. Searches for bridged bicyclic molecules in Space---Norbornadiene and its cyano derivatives. Faraday Discuss. 2023, 10.1039/d3fd00016h. PMC10510035 37305958

