# Supplementary material for: Multiconfigurational Calculations and Photodynamics Describe Norbornadiene Photochemistry
Source: J Org Chem. 2023 Apr 6;88(9):5311–20. doi: 10.1021/acs.joc.2c02758 (PMC10629221; doi:10.1021/acs.joc.2c02758)
Supplement: Supplementary file 1 — jo2c02758_si_001.pdf [file jo2c02758_si_001.pdf]

Supporting Information

Multiconfigurational calculations and photodynamics describe norbornadiene photochemistry

Federico J. Hernández,[b] Jordan M. Cox,[a] and Jingbai Li, [c,*] Rachel Crespo-Otero,[b,*] and Steven A. Lopez[a,*]

[a] Department of Chemistry and Chemical Biology, Northeastern University, Boston, MA 02115, U.S.A.

[b] School of Physical and Chemical Sciences, Queen Mary University of London, Mile End Road, London E1 4NS, U.K.

[c] Hoffmann Institute of Advanced Materials, Shenzhen Polytechnic, 7098 Liuxian Blvd, Nanshan District, Shenzhen, 518055, People's Republic of China

* Correspondence to lijingbai@szpt.edu.cn, r.crespo-otero@qmul.ac.uk, and s.lopez@northeastern.edu

Contents

S1. Additional data of the gas-phase minimum energy path of NBD S1

S2. Additional information on NBD and DCNBD trajectories...S2

S3. Electronic energies...S4

S4. Cartesian coordinates of optimized structures S4

S1. Additional data of the gas-phase minimum energy path of NBD

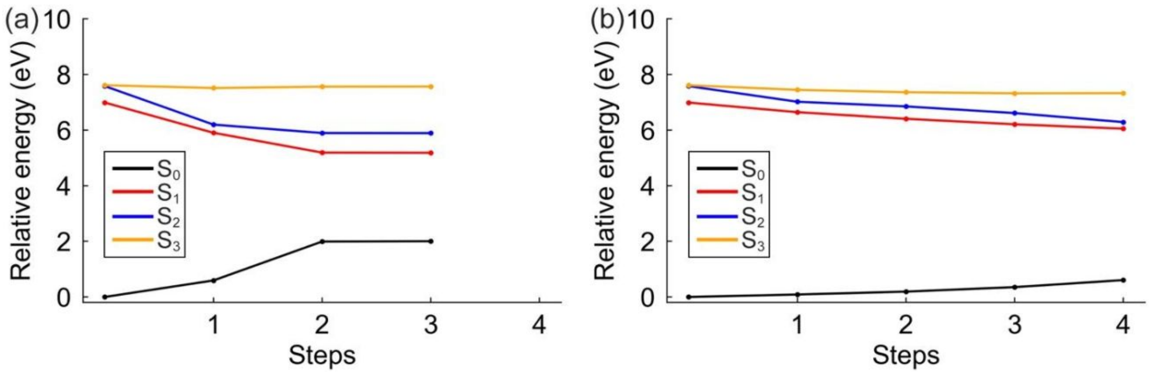

Figure S1. Minimum energy paths of NBD following the (a) S_2 and (b) S_3 states, computed with the SA6-CASSCF(4,7)/ANO-S-VDZP.

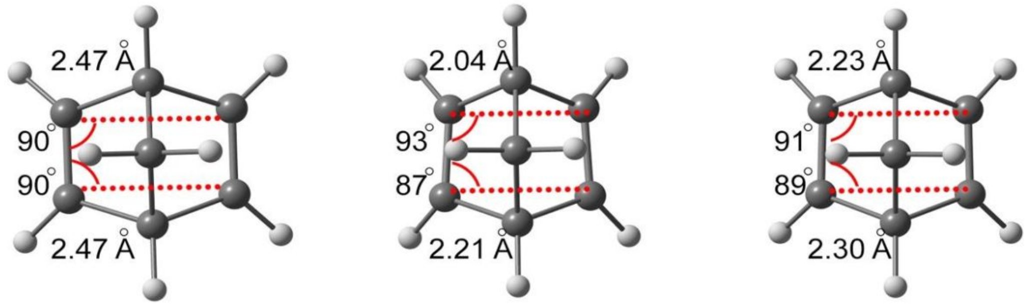

	S_1-FC	S_2-min	S_3-min
S_1	6.99	5.33	6.10
S_2	7.58	5.87	6.42
S_3	7.61	7.45	7.31

Figure S2. The geometries of NBD at the FC-S_1, S_2 and S_3 minimum, optimized with SA6-CASSCF(4,7)/ANO-S-VDZP in gas phase. The excited-state energies are in eV.

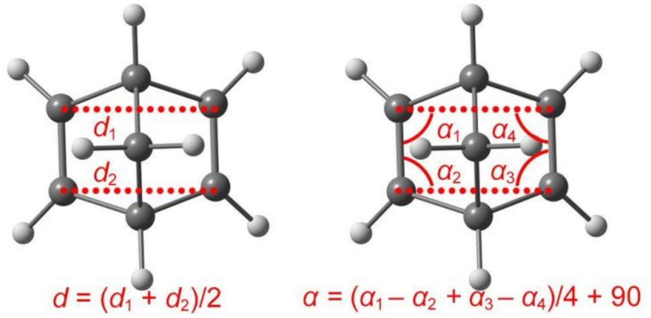

Figure S3. Definition of the distance between the $\pi_{C\text{-}C}$ bonds, *d*, and the rhomboidal angle among the bond-forming carbon, *α* in NBD.

S2. Additional information on NBD and DCNBD trajectories

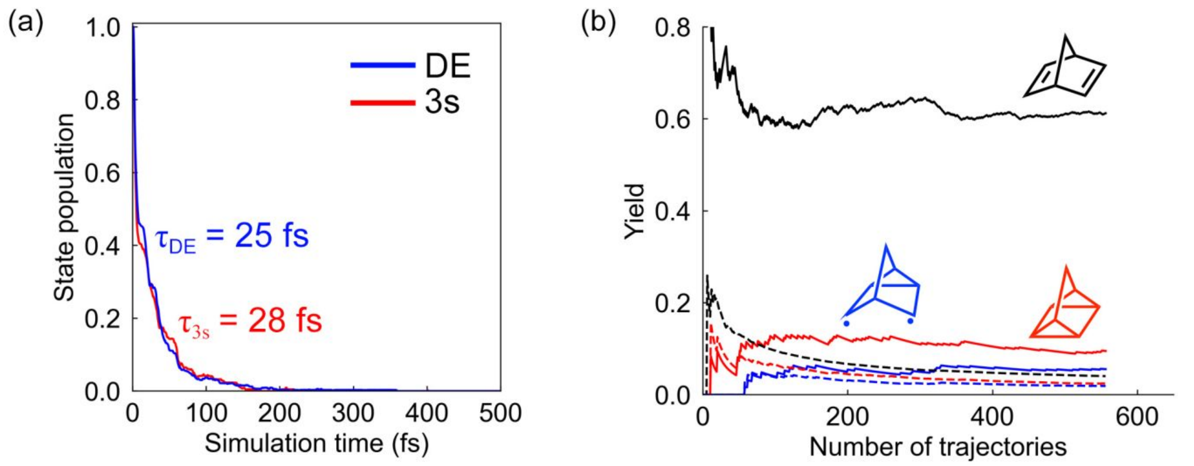

Figure S4. (a) S_2 population decay in the gas phase NBD trajectories. The populations are averaged from the trajectories being excited to the 3s-Rydberg state and doubly excited $\pi\pi^*$ state (DE), respectively. (b) Plots of the predicted quantum yields of NBD, QC, and BR as a function of the number of trajectories. The predicted values converge with 577 gas-phase trajectories. The dash lines represent the margin of error (MOE) at a 95% confidence level. The final MOE for NBD, QC, BR are 2%, 4%, and 2%.

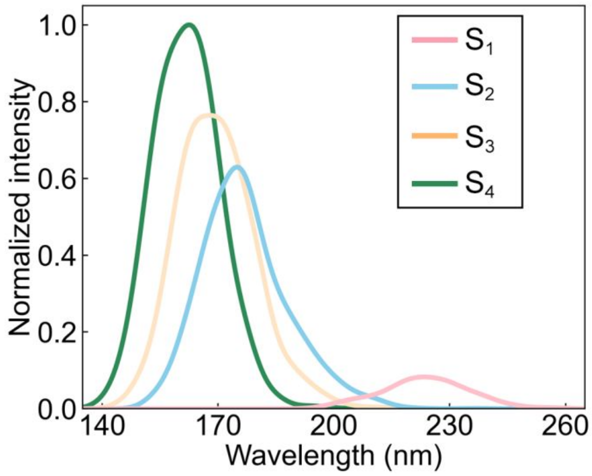

Figure S5. The simulated absorption spectrum for the first four bands of DMDCNBD. 800 structures were used to compute vertical excitation energies with SA6-CASSCF(8,6)/ANO-S-VDZP.

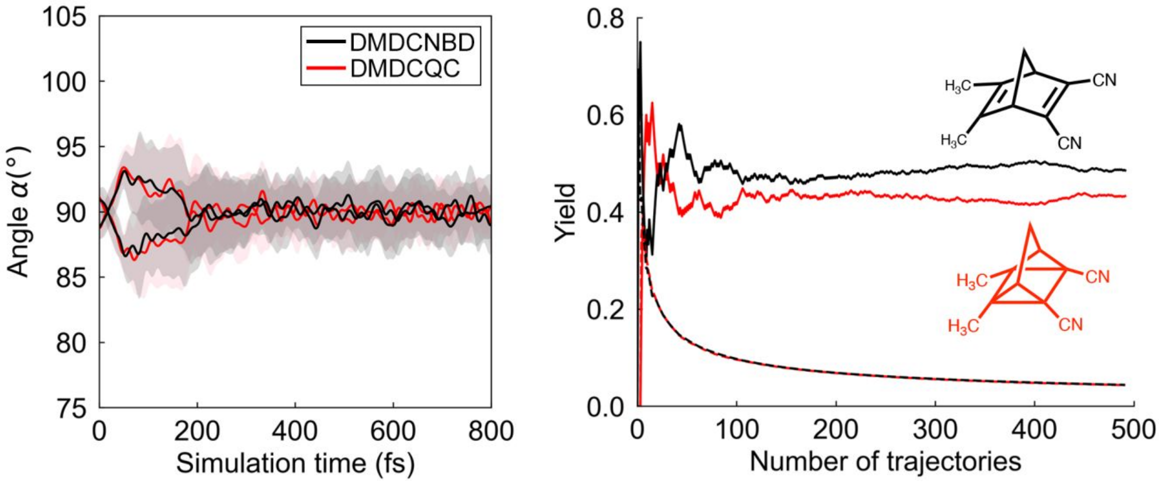

Figure S6. (a) Average rhomboidal angle of DMDCNBD trajectories. (b) Predicted quantum yields of DMDCNBD and DMDCQC as a function of the number of trajectories. The predicted values converge with 492 gas-phase trajectories. The dash lines represent the margin of error (MOE) at a 95% confidence level. The final MOE for DMDCNBD, DMDCQC are 4% and 4%.

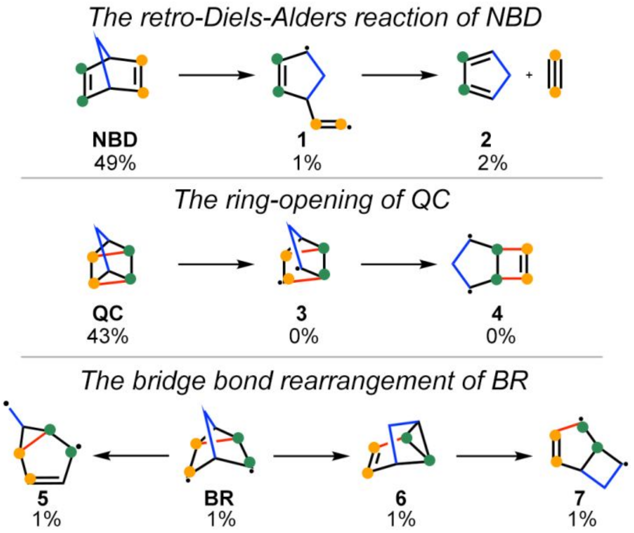

Figure S7. Side-reactions observed in the gas-phase DMDCNBD trajectories with the ratios of products or intermediates. The methylene bridge and the newly formed σ_{CC}-bonds in QC are highlighted in blue and red, respectively. The green dots denote the methyl-substituted carbon atoms, the orange dots mark the cyano-substituted carbon atoms.

S3. Electronic energies

Table S1. The electronic energy of NBD in the gas phase (g) computed at SA6-CASSCF(4,7)/ANO-S-VDZP and XMS(6)-CASPT2(4,7)/ANO-S-VDZP level.[a]

	S_0	S_1	S_2	S_3	S_4	S_5
			SA6-CASSCF(4,7)/ANO-S-VDZP			
S_0 Min	-269.74250346	-269.48567045	-269.46393101	-269.46276222	-269.44256409	-269.41322778
S_2/S_1 MECI	-269.70986170	-269.52399548	-269.52399411	-269.47272972	-269.43692056	-269.38909052
S_1/S_0 MECI	-269.58298058	-269.58297912	-269.43632398	-269.40188060	-269.40071834	-269.37169003
			XMS(6)-CASPT2(4,7)/ANO-S-VDZP			
S_0 Min	-270.63481938	-270.43265341	-270.38934470	-270.36340189	-270.35184311	-270.33812852
S_2/S_1 MECI	-270.60761353	-270.46796001	-270.41787751	-270.37566960	-270.33754462	-270.32990442
S_1/S_0 MECI	-270.52124437	-270.48555084	-270.34841615	-270.34645308	-270.32351780	-270.30604137

[a]Energies are in Hartree.

Table S2. The electronic energy of DMDCNBD, computed at SA6-CASSCF(8,6)/ANO-S-VDZP and XMS(6)-CASPT2(8,6)/ANO-S-VDZP level.[a]

	S_0	S_1	S_2	S_3	S_4	S_5
SA6-CASSCF(8,6)/ANO-S-VDZP						
S_0 Min	-531.32356764	-531.32356764	-531.32356764	-531.32356764	-531.32356764	-531.32356764
S_1/S_0 MECI	-531.17514427	-531.17514427	-531.17514427	-531.17514427	-531.17514427	-531.17514427
XMS(6)-CASPT2(8,6)/ANO-S-VDZP						
S_0 Min	-533.04501339	-533.04501339	-533.04501339	-533.04501339	-533.04501339	-533.04501339
S_1/S_0 MECI	-532.94877760	-532.94877760	-532.94877760	-532.94877760	-532.94877760	-532.94877760

[a]Energies are in Hartree.

S4. Cartesian coordinates of optimized structures

NBD S_0min

C	-0.245940016	-0.953221071	0.580985040
C	0.786277058	-0.074737005	1.299218096
C	-1.482374104	-0.077365005	0.331532023
C	0.361887027	-0.903224065	-0.841301061
H	-0.435656029	-1.922007139	1.020846076
C	1.158568085	0.886274063	0.423512030
H	1.102150080	-0.176356012	2.333683167
C	-1.108917077	0.883040065	-0.544694038
H	-2.447861176	-0.181807014	0.817014059
C	0.371170029	0.646371044	-0.871539064
H	1.356632097	-1.342817099	-0.899586067
H	-0.287045023	-1.343266095	-1.600342117
H	1.830488129	1.716266125	0.607527042
H	-1.708559124	1.712485122	-0.902512063
H	0.748576052	1.134249083	-1.755632129

NBD S_2/S_1-MECI

C	-0.23878076	-0.94695835	0.51070701
C	0.64415009	0.03409205	1.24420072
C	-1.37056253	0.04224855	0.36745587
C	0.37874721	-0.94438585	-0.90821300
H	-0.45711235	-1.88623068	1.00360797
C	1.05195052	0.97902515	0.31943593
H	0.80696068	0.04075649	2.30853635
C	-0.96536021	0.98714493	-0.55848083
H	-2.26031546	0.05315741	0.97377408
C	0.42551131	0.59969995	-1.00127861
H	1.36651587	-1.39823887	-0.93820562
H	-0.27556044	-1.39157736	-1.65284032
H	1.60308062	1.88560726	0.50300850
H	-1.46917610	1.89793746	-0.83402129
H	0.81535857	1.07546187	-1.89261478

NBD S_1/S_0-MECI

C	-0.23903861	-0.86785186	0.55024125
C	0.59688017	0.13084288	1.33389945
C	-1.24815949	0.22706900	0.51994166
C	0.35503985	-0.84296054	-0.86478956
H	-0.49791456	-1.81571293	1.00513730
C	0.61081023	1.21098277	0.29524408
H	0.28821075	0.38652719	2.34095613
C	-1.12695145	1.06120438	-0.71897569
H	-1.97238228	0.37798016	1.30549394
C	0.30368181	0.67833964	-1.06094943
H	1.36861454	-1.24164788	-0.87743835
H	-0.26297782	-1.35979922	-1.59761656
H	0.82026369	2.25300033	0.48149464
H	-1.38035557	2.11334535	-0.66025692
H	0.77898841	1.12293818	-1.92618064

NBD S_2min

C	-0.23606667	-0.95963342	0.50364360
C	0.55111741	0.07154995	1.26021830
C	-1.32282392	0.07173219	0.44588218
C	0.37825369	-0.92538430	-0.90606530
H	-0.44841734	-1.90417488	0.99036125
C	1.05158806	0.99840661	0.32413083
H	0.62772210	0.12981239	2.33318266
C	-0.97799966	1.00181764	-0.55690608
H	-2.17018504	0.11328744	1.11062698
C	0.42093127	0.61775020	-0.99429073
H	1.36798355	-1.37620084	-0.93713211
H	-0.27540467	-1.37299468	-1.65146534
H	1.58720282	1.90852201	0.53699832
H	-1.47739620	1.93232866	-0.76840214
H	0.81406631	1.08113562	-1.89155751

NBD S_3min

C	-0.24649547	-0.91116423	0.52882335
C	0.65584361	0.05084137	1.26023660
C	-1.39013812	0.05911881	0.36986223
C	0.36677725	-0.89674491	-0.88026414
H	-0.46732577	-1.85351084	1.02750337
C	1.09455500	0.97574347	0.34586633
H	0.81533884	0.07477677	2.33440174
C	-1.01373272	0.98427157	-0.57162510
H	-2.28470850	0.08732452	0.98532278
C	0.41139555	0.64141094	-0.96850552
H	1.35839300	-1.35135974	-0.91032994
H	-0.29010876	-1.34470017	-1.62773785

H	1.69292585	1.85299894	0.55802954
H	-1.57081715	1.86620744	-0.86228790
H	0.80411619	1.11536675	-1.86652747

DMDCNBD S_0min

C	-0.26854569	-0.84430290	0.58384986
C	0.73111278	0.10007644	1.31700599
C	-1.50204301	0.01776507	0.27152398
C	0.41567761	-0.86573993	-0.79596543
H	-0.44052176	-1.78307722	1.09754719
C	1.12353847	1.01687674	0.40894918
C	-1.10842953	0.93708187	-0.63896809
C	0.37949445	0.66946994	-0.91567608
H	1.42032871	-1.28238295	-0.78241871
H	-0.19272105	-1.35517154	-1.55302414
H	0.80931997	1.13636565	-1.79432720
C	1.05853564	-0.01352031	2.71317281
N	1.27564503	-0.15676101	3.81170354
C	1.98751648	2.15657840	0.56398214
N	2.65487177	3.06530260	0.62115794
C	-2.79873537	-0.14548428	1.00886144
H	-3.55345834	0.54507361	0.64307714
H	-3.18060074	-1.16108433	0.89683788
H	-2.65649008	0.03754780	2.07492273
C	-1.83265036	2.11092380	-1.22996858
H	-1.80844639	2.06973311	-2.31977223
H	-2.87122883	2.13803998	-0.91237238
H	-1.35797477	3.04452448	-0.92469498

DMDCNBD S_1/S_0-MECI

C	-0.11743733	-1.14071854	0.41337309
C	1.05298418	-0.36926495	1.09538122
C	-1.00482878	0.01906095	0.60527119
C	0.26046505	-1.07788730	-1.07322960
H	-0.41771031	-2.08004331	0.85679997
C	0.92869748	0.81357528	0.19044368
C	-0.79988826	1.01742098	-0.40010147
C	0.40561610	0.43978522	-1.14043483
H	1.19018893	-1.60418127	-1.26534113
H	-0.52578762	-1.43229086	-1.73788602
H	0.76197968	0.94734571	-2.02388099
C	0.99280293	-0.16739190	2.50575408
N	0.98226822	-0.04878018	3.64486161
C	1.53593795	2.10969910	0.40599050
N	2.00889495	3.13023615	0.55212330
C	-1.97223634	0.13145767	1.72660700
H	-1.70622034	-0.52320145	2.54600382
H	-2.07452189	1.15190594	2.07819463

H	-2.93127320	-0.18750885	1.30466167
C	-1.42195946	2.37976864	-0.52747846
H	-2.36802557	2.32929382	-1.07012367
H	-1.60684294	2.83994157	0.44041710
H	-0.76022251	3.04254563	-1.07971924
